# Targeting Histone Deacetylases in Idiopathic Pulmonary Fibrosis: A Future Therapeutic Option

**DOI:** 10.3390/cells11101626

**Published:** 2022-05-12

**Authors:** Martina Korfei, Poornima Mahavadi, Andreas Guenther

**Affiliations:** 1 Biomedical Research Center Seltersberg (BFS), Justus Liebig University Giessen, D-35392 Giessen, Germany; poornima.mahavadi@innere.med.uni-giessen.de (P.M.); andreas.guenther@innere.med.uni-giessen.de (A.G.); 2 Department of Internal Medicine, Universities of Giessen and Marburg Lung Center (UGMLC), Member of the German Center for Lung Research (DZL), D-35392 Giessen, Germany; 3 Lung Clinic, Evangelisches Krankenhaus Mittelhessen, D-35398 Giessen, Germany; 4 European IPF Registry and Biobank, D-35392 Giessen, Germany

**Keywords:** idiopathic pulmonary fibrosis (IPF), histone deacetylase (HDAC), histone acetylation, non-histone protein acetylation, fibroblast-to-myofibroblast differentiation (FMD), type-II alveolar epithelial cell (AECII), bronchiolar basal cells, bronchiolization, Class I-HDAC-inhibitor, (pan-)HDAC-inhibitor

## Abstract

Idiopathic pulmonary fibrosis (IPF) is a progressive and fatal lung disease with limited therapeutic options, and there is a huge unmet need for new therapies. A growing body of evidence suggests that the histone deacetylase (HDAC) family of transcriptional corepressors has emerged as crucial mediators of IPF pathogenesis. HDACs deacetylate histones and result in chromatin condensation and epigenetic repression of gene transcription. HDACs also catalyse the deacetylation of many non-histone proteins, including transcription factors, thus also leading to changes in the transcriptome and cellular signalling. Increased HDAC expression is associated with cell proliferation, cell growth and anti-apoptosis and is, thus, a salient feature of many cancers. In IPF, induction and abnormal upregulation of Class I and Class II HDAC enzymes in myofibroblast foci, as well as aberrant bronchiolar epithelium, is an eminent observation, whereas type-II alveolar epithelial cells (AECII) of IPF lungs indicate a significant depletion of many HDACs. We thus suggest that the significant imbalance of HDAC activity in IPF lungs, with a “cancer-like” increase in fibroblastic and bronchial cells versus a lack in AECII, promotes and perpetuates fibrosis. This review focuses on the mechanisms by which Class I and Class II HDACs mediate fibrogenesis and on the mechanisms by which various HDAC inhibitors reverse the deregulated epigenetic responses in IPF, supporting HDAC inhibition as promising IPF therapy.

## 1. Introduction

### 1.1. Pathomechanisms of Idiopathic Pulmonary Fibrosis

Idiopathic pulmonary fibrosis (IPF) is a devastating interstitial lung disease of unknown origin with a poor prognosis. It predominantly affects individuals aged 60 to 75 years old, with a median mortality rate of 3–5 years after diagnosis, which is comparable to or even worse than many cancers [1,2]. Although pirfenidone (Esbriet^®^) and nintedanib (Ofev^®^) have recently been approved as IPF therapies, which are effective in reducing the rate of lung function decline, neither is curative for the disease [3,4,5]. IPF still has a high mortality rate, and there is an unmet medical need for an improved drug or for a cure.

The current pathogenic model of IPF suggests that lung fibrosis develops as a result of unremitting insults in combination with genetic- and ageing-related risk factors to type-I/-II alveolar epithelial cells (AECI/II), which consecutively trigger an aberrant wound healing response through the activation of fibroblasts and myofibroblasts and the replacement of injured alveolar epithelium with fibrotic scar tissue due to a decreased renewal capacity of the alveolar epithelium [6,7,8]. The so-called fibroblast foci, subepithelially located, represent the active sites of fibrosis and consist of apoptosis-resistant myofibroblasts and the extracellular matrix (ECM) they produce, resulting in persistent collagen deposition, progressive scarring and overall lung tissue stiffness [6,9]. Another prominent hallmark of IPF is the bronchiolisation of distal alveoli, involving structures that are composed of “proliferative” bronchiolar basal cells and mucin-producing airway secretory cells [10,11,12]. In addition, it has been widely observed that airway epithelium consisting of p63^+^ cytokeratin-5/KRT5^+^ positive basal cell sheets (underneath luminal-ciliated bronchial cells) overlie the fibroblast foci, indicating that the integrity of the alveolar epithelium is severely disrupted in IPF [10,13]. In agreement, death of AECII is a prominent feature in IPF [8,14,15,16,17] and has been linked to endoplasmic reticulum (ER) stress as a variety of studies have documented the induction of the unfolded protein response (UPR) and markers of pro-apoptotic ER stress in the AECII of patients with sporadic and familial IPF [18,19,20,21].

#### 1.1.1. Genetic Factors Affecting IPF-Epithelial Cells

Compelling evidence indicates that genetic susceptibility plays a part in AECII ER stress and the development of IPF. Among the stimuli and triggering conditions capable of inducing the UPR and ER stress in AECII are the discovered (heterozygous) mutations in the surfactant protein (SP)-C (*SFTPC*)- and SP-A2 (*SFTPA2*) genes in familial IPF, which cause misfolded SP-C and SP-A2 proteins, respectively [22,23,24,25]. In experimental models with transgenic mice that conditionally overexpress the mutation *SFTPC*^C121G^ in AECII, ER stress severely increased after the induction of mutant SP-C^C121G^ protein expression, which resulted in AECII apoptosis and the development of spontaneous lung fibrosis in mice [26], suggesting that AECII ER stress indeed precedes the development of fibrosis in human IPF. Importantly, ER stress and apoptosis do not seem to differ in extent between *SFTPC*/*A2*-associated familial IPF and sporadic IPF cases in the absence of gene mutations [19]. In addition, mutations in six genes linked to telomere function have been found in familial IPF (telomerase reverse transcriptase, *TERT* [27,28]; telomerase RNA component, *TR* [27,28]; dyskerin, *DKC1* [29]; telomere interacting factor 2, *TINF2* [30]; regulator of telomere elongation helicase, *RTEL1* [31]; and poly(A)-specific ribonuclease deadenylation nuclease, *PARN* [31]), which implicate telomere shortening and DNA-damage responses in IPF pathogenesis and which are also strongly suggested to induce AECII apoptosis [27,28,29,31]. It was shown that systemic telomere attrition in AECII, but not fibroblasts, led to lung remodelling and fibrosis in a mouse model [32]. However, it is currently unclear which role ER stress may play under these conditions and *vice versa*; it is currently unknown why AECII ER stress is a prominent characteristic of sporadic cases of IPF [18], which comprise ~85% of the total IPF population [33]. Importantly, premature telomere shortening has also been observed in the AECII of sporadic IPF patients [34]. Moreover, some of these abovementioned mutations have been reported not only in familial IPF but also in sporadic IPF cases [34], suggesting a similarity between these two types of IPF and that sporadic IPF is also a disease with a genetic predisposition. In agreement, genome-wide association studies have found that a single-nucleotide polymorphism (SNP) in the promoter region of the mucin 5B gene is the strongest risk factor for familial and sporadic IPF described so far as this gain-of-function *MUC5B* promoter variant rs35705950 was similarly present in subjects with familial and sporadic IPF [35,36], accounting for 30–35% of the risk of developing IPF [35]. Interestingly, the rs35705950 variant not only predisposes to IPF but has also been associated with improved survival compared with patients without this variant, although this latter association remains somewhat controversial because this gain-of-function variant was shown to result in increased mucin 5B expression and impaired mucuciliary clearance in the bronchial cells of IPF subjects (as well as healthy subjects) carrying this variant, suggesting that bronchial cell defects can affect the onset of disease [37]. Moreover, further research by the same group revealed that *MUC5B* was even found to be co-expressed with *SFTPC* expression in the columnar epithelial cells of abnormal bronchiolar structures as well as the AECII of IPF patients with the rs35705950 variant, but also in normal bronchioles and AECII of healthy subjects with this variant [38]. It was also demonstrated that transgenic mice overexpressing Muc5b in the distal lung indicated greater and more aggravated lung fibrosis than wild-type mice following bleomycin treatment [38]. With this conflicting background, the functional and pathomechanistic consequences of the *MUC5B* rs35705950 T/G polymorphism in IPF need further investigation.

#### 1.1.2. The Core in IPF: Disturbed AECII–Mesenchymal Communication and AECII/Fibroblast Apoptosis Imbalance

Considering IPF as an alveolar epithelium-driven disease, the molecular mechanisms leading to fibrotic remodelling and the aberrant epithelial repair, including the abnormal bronchiolisation process in response to the causal AECII injury and death, are still incompletely resolved. Following injury, AECII proliferates and differentiates into AECI for the repair of alveolar structure [38]. In IPF, however, AECII differentiates and features an abnormally activated phenotype, characterised as cells undergoing hyperplasia, senescence and apoptosis, which contribute as paracrine factors to fibroblast proliferation and their transformation into myofibroblasts through the production and release of profibrotic cytokines and growth factors (amongst them, transforming growth factor beta (TGF-β), connective tissue growth factor (CTGF), platelet-derived growth factor (PDGF), tumour necrosis factor-alpha (TNF-α), and endothelin-1) or other mediators [39,40,41,42]. Targeted AECII damage in transgenic mice was also shown to induce plasminogen activator inhibitor 1 (PAI-1) overexpression in AECII and lung macrophages, which resulted in the development of fatal lung fibrosis. [43]. Another study revealed that the uptake of apoptotic AECII by alveolar macrophages contributed to fibrosis through the increased expression and secretion of TGF-β by such activated macrophages [44]. Aside from AECII and macrophages, TGF-β and the abovementioned profibrotic molecules are excessively found in fibroblast foci and continuously promote AECII apoptosis via autocrine and paracrine mechanisms [41,45,46]. Myofibroblasts have been shown to upregulate NADPH oxidase 4 (NOX4) to produce high levels of extracellular H_2_O_2_ in response to TGF-β (or other growth factors), which promote damage to AECII while increasing fibroblast proliferation, fibroblast-to-myofibroblast differentiation (FMD) and resistance to apoptosis in fibroblasts and myofibroblasts [47,48]. Other factors promoting the proliferation and anti-apoptosis of fibrotic fibroblasts/myofibroblasts include the increased expression of inhibitors of apoptosis in myofibroblasts, such as surviving [49], cellular FLICE-like inhibitory protein (c-FLIP) [50,51], phosphatidylinositol-3-kinase-gamma (PI3K-γ) [52], secreted protein acidic and rich in cysteine (SPARC) [53], and X-linked inhibitor of apoptosis (XIAP) [54].

#### 1.1.3. Key Fibrotic Pathways behind IPF

TGF-β has been regarded as a central factor in fibroblast activation, driving the development of lung fibrosis and IPF progression through the activation of numerous profibrotic and survival-related signalling cascades [55]. TGF-β interacts with its receptors (TGF-βRs) on the surface of fibroblasts, and, in the canonical pathway, it phosphorylates mothers against decapentaplegic homolog (SMAD)2 and 3, which then heterodimerise with SMAD4 to form SMAD2/4 and SMAD3/4 complexes that translocate to the nucleus to activate profibrotic- and proliferation-related genes [55]. In the non-canonical (SMAD-independent) activation pathways, tyrosine-protein kinase ABL1 [56], Janus kinases (JAK) [57,58], PI3K [52,59], and mitogen-activated protein kinases (MAPKs) [60] have been shown to be directly activated by TGF-β, mediating persistent activation of fibroblastic cell populations in IPF.

The contribution of the PI3K/protein kinase B (PKB/AKT) signalling pathway to both fibroblast proliferation and differentiation into myofibroblasts is prominent as fibroblasts isolated from IPF patients have been demonstrated to display pathological activation of AKT [61] and pan-inhibition of upstream class I PI3Ks by the small molecule LY294002-abrogated TGF-β-induced proliferative effects as well as α-SMA expression and collagen production in lung fibroblasts in vitro and bleomycin-induced lung fibrosis in rats in vivo [62,63]. The catalytic subunit of PI3K occurs in four isoforms (α, β, γ, and δ), which are ubiquitously expressed and found in lung fibroblasts [59]. Interestingly, selective suppression of PI3K-α or PI3K-γ by *small interfering* (si)RNAs was able to elicit significant antifibrotic effects in TGF-β-stimulated human lung fibroblasts, comparable to that induced by pan-PI3K inhibition, confirming a crucial role of these both isoforms in fibrotic lung fibroblasts [62]. Moreover, PI3K-γ has been found to be significantly overexpressed in fibroblast foci and bronchiolar basal cells in IPF lungs and co-localised with cell markers for proliferation and survival [52]. Subsequent research revealed that targeting PI3K-γ activity genetically or pharmacologically (by the small molecule AS-252424 or AS-605240) was able to significantly dampen fibrogenesis in IPF-fibroblast cultures alone and prevent bleomycin-induced lung fibrosis in rats in vivo [52,64]. These findings are important, considering that in cancer, the activation of the PI3K-γ pathway is involved in the lack of regulation of cell proliferation [65]. Moreover, other studies indicate that the increase of AECII apoptosis in IPF is closely related to pathological PI3K/AKT activation, which causes the release of H_2_O_2_ and subsequent damage to adjacent AECIIs [66,67].

Several studies also indicate a significant role of phosphorylated, activated signal transducer and activator of transcription 3 (STAT3), which can be induced by TGF-β, PDGF, as well as the IL-6 family of cytokines in IPF fibroblasts [68,69,70]. In response to such ligands, STAT3 becomes specifically phosphorylated at tyrosine 705 (Tyr705) by growth factor/cytokine receptor-associated JAK2 kinase, translocates to the nucleus and serves as a potent transcription factor for surviving [71] and the genes involved in myofibroblast differentiation [70]. In IPF fibroblasts, active p-STAT3 was shown to confer resistance to FasL-induced apoptosis [72]. It could also be demonstrated that C-188-9, a small molecule STAT3 inhibitor that targets the Tyr705 peptide binding pocket, decreased FMD induced by TGF-β in cultured lung fibroblasts as well as significantly reduced experimental pulmonary fibrosis in mice [69]. Similarly, selective JAK2 tyrosine kinase inhibition by fedratinib attenuated TGF-β- and IL-6-induced myofibroblast activation regulated by JAK2/p-STAT3 as well as reduced bleomycin-induced lung fibrosis in mice in vivo [70]. Interestingly, it was shown that STAT3 phosphorylation participates in both lung epithelial damage and fibroblast-to-myofibroblast transformation [69]. Studies also revealed that JAK2/STAT3 signalling undergoes hyperactivation in IPF patients [73].

Enhanced activation of the coagulation cascade, including the significant overexpression of several zymogens in the alveolar compartment, as well as fibroblast foci, has also been demonstrated in the setting of pulmonary fibrosis and IPF [74]. In addition to fibrin deposition in the lungs, it has been shown that this cascade is also closely related to ECM generation as locally produced and circulation-derived FII (thrombin) and/or FXa were shown to induce profibrotic effects via the proteolytic activation of protease-activated receptor-1 (PAR1) and the subsequent differentiation of fibroblasts into myofibroblast [75,76]. Thrombin was also shown to induce CTGF expression in human lung fibroblasts through the activation of the c-Src/JAK2/STAT3 signalling pathway [77]. *Vice versa*, in AECII, thrombin induced cell death through the induction of pro-apoptotic ER stress [78]. In aggregate, in IPF, the dysregulated crosstalk and the abnormally increased profibrotic mediators between AECII and fibroblasts lead to AECII apoptosis, fibroblast anti-apoptosis, excessive ECM deposition and aberrant bronchiolar tissue generation.

#### 1.1.4. Deregulation of microRNAs in IPF

The deregulation of microRNAs (miRNAs) in pulmonary fibrosis has also received much attention as it contributes to the evolution and progression of the disease [79]. miRNAs are non-coding RNAs, 18–22 nucleotides in length, that repress gene expression by decreasing stability or inhibiting the translation of target messenger RNAs. Various miRNA microarray analyses showed that the expression of profibrotic miR-21 and miR-199a-5p was increased in the lungs of IPF patients as well as bleomycin-injured mice, while the expression of anti-fibrotic miR-26a, let-7d, miR-9-5p, miR-29 and miR-200 was decreased [79,80,81]. SMAD7, which is known to inhibit TGF-β/SMAD2/3 signalling, is a direct target of miR-21, and the upregulation of miR-21 promoted cell proliferation and collagen synthesis in lung fibroblasts [82,83]. The upregulation of miR-199a-5p during the fibrotic response to epithelial injury mediated TGF-β induced fibroblast activation through the degradation of the anti-fibrotic mediator caveolin-1 [84]. Among the anti-fibrotic miRNAs downregulated in lung fibrosis, miR-26a was shown to inhibit myofibroblast differentiation and experimental lung fibrosis through its ability to downregulate the expression of its target CTGF [85], and forced expression of miR-9-5p was demonstrated to suppress FMD and lung fibrosis development through downregulating Nox4 and TGF-βRII [86]. Similarly, miR-29 was reported as a main negative regulator of ECM production [87]. Decreased expression of let-7d and miR-200 has been associated with the abnormally activated phenotype of AECII in IPF [81,88], whereas the overexpression of miR-200 family members was recently demonstrated to reduce senescence in primary IPF-AECII in vitro and restore their ability to transdifferentiate into AECI [89]. Moreover, antagomirs for the augmentation of miR-323a-3p, which is also found downregulated in the epithelial cells of IPF lungs, were shown to lower epithelial caspase-3 expression and TGF-β signalling and suppress murine lung fibrosis after bleomycin injury [90]. Taken together, cell-specific deregulation of miRNAs significantly contributes to AEC/fibroblast apoptosis imbalance and the production of profibrotic mediators in IPF.

### 1.2. Treatment of Idiopathic Pulmonary Fibrosis

#### 1.2.1. Established Therapies for IPF

At present, the FDA-approved drugs nintedanib (Ofev^®^, Boehringer Ingelheim, Ingelheim, Germany) and pirfenidone (Esbriet^®^, Roche, Basel, Switzerland) are widely used for IPF therapy [91,92]. Nintedanib is a small molecule nonreceptor and receptor tyrosine kinase inhibitor. Nintedanib can block activation of platelet-derived growth factor receptor (PDGFR), fibroblast growth factor receptor (FGFR), vascular endothelial growth factor receptor (VEGFR) and Src family kinases involved in fibroblast proliferation, migration, and transformation [93]. The antifibrotic property of pirfenidone is demonstrated by its ability to inhibit the direct production of profibrotic growth factors and cytokines, such as TGF-β, interleukin-1β (IL-1β), and TNF-α [94,95,96,97], as well as the synthesis of procollagens I and II [98,99]. Pirfenidone has also been reported to attenuate experimental lung fibrosis through the reduction of reactive oxygen species (ROS) generation by downregulating Nox4 expression and improving the expression of antioxidant enzymes such as superoxide dismutase (SOD), catalase and glutathione peroxidase (GPx1) [100,101].

The efficacy and safety of nintedanib and pirfenidone have been demonstrated in several large phase 3, randomised, controlled clinical trials in patients with IPF [3,4,102,103]. It has also recently been proven that combining both drugs indicates controllable safety and tolerability in patients [104], but the efficacy is still under evaluation in trials. Whether the combination of pirfenidone and nintedanib may enhance efficacy is questionable. It has been reported that both drugs simply mitigate symptoms and retard progression but fail to significantly prolong survival [5]. Thus, IPF research is increasingly focused on developing new molecular targets and treatment options.

#### 1.2.2. Therapeutic Targets Proposed for IPF Treatment

Agents that inhibit the activation of TGF-β signalling are currently under study. The integrin alpha-V:beta-6 (αvβ6 integrin) is known as a key driver of TGF-β activation [105], and it is significantly upregulated in IPF-lung tissue and localised to damaged epithelial sites [106]. The development of a monoclonal antibody against αvβ6 integrin (clinically known as BG00011) has completed a phase 2 trial for IPF (clinicaltrials.gov (accessed on 20 January 2022) identifier NCT01371305). BG00011 was shown to suppress TGF-β activation in IPF patients, as evidenced by a reduction of p-SMAD2 signalling and TGF-β dependent gene expression in the BAL cells of patients. However, BG00011 was withdrawn due to safety concerns [107]. In contrast to BG00011, the small molecule αvβ6 integrin-inhibitor GSK3008348 developed by GlaxoSmithKline represents a therapeutic agent for inhaled delivery to IPF patients. It was found to efficiently degrade αvβ6 integrin in IPF tissues and isolate epithelial cells from IPF patients [108]. Although a phase 1 first-time-in-humans clinical trial (NCT02612051) revealed that inhaled GSK3008348 was safe and well-tolerated [108], the study also recently underwent early termination. Currently, a phase 2a randomised, double-blind, dose-ranging, placebo-controlled study of a dual selective inhibitor of the integrins αVβ1/αVβ6 (clinically known as PLN-74809) in IPF is ongoing (NCT04396756). Another approach to target TGF-β signalling is via gene silencing, as exemplified by TRK-250, a single-stranded oligonucleotide that produces siRNA targeting human *TGFB* mRNA. A phase 1 study of this inhaled nucleic acid medication is currently in progress (NCT03727802).

As described above, the PI3K/AKT pathway has been demonstrated to offer a reasonable target for the treatment of IPF [59]. Despite the evident antifibrotic effects of the specific PI3K/AKT inhibitor LY294002 in preclinical models of lung fibrosis [62,63], clinical trials with this drug have yet not been initiated. Further, there has been no new progress in the use of other pan- or isoform-specific PI3K inhibitors in the clinical treatment of IPF.

Because the JAK2/STAT3 signalling pathway is crucially involved in IPF [58,70,73], inhibitors targeting this pathway have been proposed to treat the disease. However, there are no clinical trials that study STAT3 inhibition in IPF patients. Further, the clinical use of JAK inhibitors is only described in myelofibrosis and autoimmune-disorder-associated interstitial lung disease (ILD) [109,110], and there is still a lack of studies on idiopathic ILDs. The abovementioned JAK2 inhibitor fedratinib [70], sold under the brand name Inrebic, is an approved anti-cancer medication used to treat myelofibrosis and other myeloproliferative diseases [109]. Baricitinib (Olumiant), a small molecule JAK1/JAK2 inhibitor, is approved for the treatment of rheumatoid arthritis (RA) and has been proven to reduce lung fibrosis and inflammation in patients with RA-associated ILD (RA-ILD) [110]. The JAK1/2/3 inhibitor tofacitinib (Xeljanz) is another approved therapy for RA [111]. Ruxolitinib (Jakafi) is another JAK1/JAK2 inhibitor approved for the treatment of intermediate and high-risk myelofibrosis [112]. This drug has also been shown to significantly ameliorate bleomycin-induced lung fibrosis in mice [113]. Tocilizumab (Actemra), a humanised monoclonal antibody against the interleukin-6 receptor, acts as an indirect JAK/STAT inhibitor through the inhibition of IL-6/JAK/STAT signalling and is used for the treatment of moderate-to-severe RA [114]. Tocilizumab was also granted emergency use authorization (EUA) for the treatment of Coronavirus Disease 2019 (COVID-19) in the United States in June 2021 [115].

Despite their significant therapeutic effects on myofibroblast activation in preclinical models of lung fibrosis [70,73,113], JAK inhibitors have yet not been evaluated for the treatment of IPF, presumably due to toxicities related to their immunosuppressive effects, including infectious events [116].

#### 1.2.3. Senotherapies for IPF

Very recently, senolytics have been suggested as IPF therapy [117]. AECII senescence is a hallmark in IPF that is suspected of driving lung fibrosis as a paracrine factor through the senescence-associated secretory phenotype (SASP), involving the release of multiple profibrotic cytokines and ROS molecules [7,118,119]. A recent study demonstrated that the combination of quercetin, a natural compound with antioxidant properties, and the tyrosine kinase inhibitor dasatinib attenuated progressive lung fibrosis in a transgenic mouse model with conditional p53/p21-induced AECII senescence through the ablation of senescent AECII [120]. In addition, this senolytic cocktail was also shown to attenuate bleomycin-induced lung fibrosis in mice [121]. Importantly, quercetin is also described as a potent inhibitor of NOX4 [122], which is found upregulated in fibroblast foci as well as the AECII of IPF lungs [47,48], and the full deficiency of *Nox4* has been shown to protect mice against bleomycin-induced AECII apoptosis and lung fibrosis [123]. Moreover, another study suggested that quercetin abrogates the resistance to apoptosis in IPF fibroblasts via the up-regulation of FAS and caveolin-1 and the inhibition of AKT phosphorylation [124]. Based on the therapeutic effects of the senolytic drug combination quercetin and dasatanib in preclinical data, a first-in-humans, small scale, pilot clinical trial for this senolytic cocktail has been undertaken in stable IPF patients, which was generally well-tolerated in patients. Although the effects of both senolytics on circulating SASP-factors were inconclusive, patients showed improved physical function [125]. However, evaluation of drug combination quercetin and dasatanib in larger randomised controlled trials for IPF has yet not been initiated. It should also be noted that each quercetin and dasatanib act on a myriad of pathways and mechanisms implicated in diverse biological processes, which makes it difficult to decipher how they eliminate or otherwise impact senescent cells. Thus, it is difficult to attribute any therapeutic or detrimental effects they may have on senescent cells. Further, senescent cells per se appear to be heterogeneous collections of cells with fewer shared core properties than anticipated; additionally, the composition of the SASP varies by cell type and senescence-inducing stressor [126]. It is not 100% clear if every type of senescence is actually targeted by quercetin and dasatanib. Further, dasatanib, known as the approved therapy for chronic myelogenous leukemia (CML), reveals major known adverse effects, such as pleural effusion and pulmonary arterial hypertension (PAH) [127]. We thus suggest that the use of senolytics as a therapeutic option for the treatment of IPF should be reconsidered.

#### 1.2.4. Current Therapies for IPF in Development

Current IPF therapies in (advanced) development include mainly molecules that are directed against several growth factors and cytokines or other molecular targets known to play a role in the proliferation, activation, differentiation or inappropriate survival of fibroblasts.

Lysophosphatidic acid (LPA) has been identified as a key fibroblast chemokine in experimental lung fibrosis and is believed to increase fibroblast recruitment and the apoptosis resistance of fibroblasts through the activation of the LPA_1_ receptor [128]. In line, LPA_1_ receptor knockout mice are protected from bleomycin-induced lung fibrosis [129]. Increased LPA levels have also been seen in the BALF from patients with IPF [130]. The LPA_1_ receptor antagonist, BMS-986278, has shown promise in pre-clinical and phase 1 studies [131,132] and is currently in phase 2 clinical trials, with study arms for both IPF and PF-ILD subjects (NCT0438681).

Pamrevlumab, a human recombinant monoclonal antibody against CTGF, which plays an important role in fibrosis, has been shown in the phase 2 trial PRAISE to significantly reduce the decline of FVC and progression of IPF. Pamrevlumab was well tolerated, with no significant differences from placebo in the adverse event profile [133].

Pentraxin-2 (PTX2) is a serum amyloid reported to have an antifibrotic and anti-inflammatory effect by inhibiting the differentiation of monocytes into profibrotic macrophages and fibrocytes [134]. It also inhibits the direct production of TGF-β [135]. PTX2 levels are significantly lower in lung tissue, BALF and serum of IPF-patients than in healthy subjects [135,136]. The results of a second randomised, double-blind placebo-controlled phase 2 study (PRM-151-202) of IPF patients receiving recombinant human pentraxin-2 in intravenous infusions in comparison with placebo showed a significantly slower decline in pulmonary function and improved physical capacity in the PRM-151 group. The infusions were well tolerated and had increased circulating levels of PTX2 [137]. The phase 3 efficacy and safety study of PRM-151 (NCT04552899) is underway, with an estimated completion date of December 2023.

Galectin-3 is another potential target under investigation as antifibrotic therapy in IPF. Galectin-3 is a profibrotic β-galactosidase-binding protein that is elevated in the BALF and serum of patients with IPF [138]. The profibrotic function of Galectin-3 is multifactorial due to its ability to cross-link and promote signalling via multiple cell surface receptors, including integrins and growth factor receptors, such as TGF-β, VEGF and PDGF receptors [139,140]. A phase 1/2 clinical trial with TD139, an inhaled small-molecule galectin-3 inhibitor, has revealed promising results in IPF patients and healthy subjects as TD139 was well-tolerated and patients showed reduced serum levels of biomarkers of IPF progression, including PDGF and the chemokine CCL18, compared to placebo [140].

#### 1.2.5. Past Treatment Strategies Not Recommended Anymore

Medications examined in multiple clinical studies in the past, such as anticoagulation (warfarin) [141], N-acetylcysteine in combination with either azathioprine–prednisone (PANTHER trial) [142] or pirfenidone (PANORAMA study) [143], endothelin receptor antagonists (BUILD trials, ARTEMIS-IPF trial) [144,145], phosphodiesterase inhibitors (sildenafil) [146], imatinib [147], cyclophosphamide [148], interferon gamma-1b (INSPIRE) [149], and simtuzumab (monoclonal antibody against lysyl oxidase homolog 2 [LOXL2]) [150], are not recommended anymore as IPF therapies because they are ineffective or harmful.

In conclusion, there is yet no available curative treatment for IPF. Further, it is unpredictable if therapies employing antagonists directed against individual profibrotic molecules (mentioned in Section 1.2.4.) will help to cure IPF. Therefore, there is still an unmet medical need for novel drugs or for a cure.

### 1.3. Similarities between IPF and Cancer: Histone Deacetylases as Novel Therapeutic Targets in IPF

Although main molecular pathways responsible for fibroblast activation and disease progression can be blocked by the established therapies nintedanib or pirfenidone, no cure of IPF can be achieved with these drugs, presumably due to the non-targetable irreversible “endless healing” process in IPF, which is self-perpetuating, as increased lung tissue stiffness and epithelial damage further recruits and activates myofibroblasts without any exogenous stimulus [151,152]. Further, the salient features of the progressive fibrotic phenotype of IPF fibroblasts include the resistance to apoptosis and the acquired ability of IPF fibroblasts to invade ECM [153] as well as damage the basement membrane underneath the (injured) epithelium [154]. Apoptosis resistance and the invasive phenotype appear durable because they persist in isolated IPF fibroblasts after their removal from patients [155]. In this regard, IPF resembles cancer. However, myofibroblasts within fibroblast foci in IPF are polyclonal and “disease-derived” whereas cancer cells are thought to be monoclonal. Though, IPF shares a series of risk factors (ageing, smoking and environmental exposures), pathogenic pathways (PI3K-γ/AKT, JAK2/STAT3) and biological abnormalities (genetic and epigenetic alterations) with cancer [156]. In particular, there is a growing interest in the epigenetic abnormalities characterizing IPF and cancer. Epigenetic mechanisms lead to changes in gene expression without alterations in the DNA sequence and can be mediated by the expression of non-coding RNAs, DNA methylation and histone modifications. Recent studies show that IPF and (lung) cancer share the deregulation of some miRNAs. As with IPF, the expression of (above-mentioned) miR-200 and let-7d was reported to be downregulated in various cancers [157,158,159], while miR-21 (targeting SMAD7) was upregulated and allied with high oncogenic property [160,161]. Further, similar to tumour cells, results from our group and other groups suggest that epigenetic histone modifications account for the aggressive phenotype and the persistent activated state of IPF fibroblasts [162,163,164,165], which indicated a “cancer-like” upregulation of almost all Class I and Class II histone deacetylase (HDAC) enzymes and (amongst other HDAC-induced activities) [165] the abnormal “malignant” repression of proapoptotic genes [163,164]. HDACs are enzymes that deacetylate chromatin and lead to epigenetic repression of gene transcription, whereas HDAC inhibitors favour chromatin acetylation resulting in active chromatin, facilitating gene transcription [166]. Huang et al. (2013) found that increased HDAC expression was responsible for the downregulation of the apoptosis-mediating surface antigen *FAS* [tumour necrosis factor (TNF) receptor superfamily member 6] in fibrotic fibroblasts, and treatment with HDAC inhibitors increased FAS expression and restored susceptibility to FAS-mediated apoptosis [163]. Similarly, epigenetically repressed expression of the proapoptotic *BAK* (Bcl-2 homologous antagonist/killer) gene in IPF fibroblasts was shown to be reversed in response to HDAC-inhibitor treatment [164]. In addition, defective histone acetylation was shown to be responsible for the diminished expression of the antifibrotic cyclooxygenase (COX)-2 enzyme in IPF fibroblasts, which could be restored by HDAC-inhibitor treatment [162]. Interestingly, and *vice versa*, treatment of IPF fibroblasts with HDAC inhibitors resulted in the reduction of profibrotic genes, such as *COL3A1*, in association with marked chromatin alterations [164,165,167]. Further, many scientific groups have demonstrated that various HDAC inhibitors targeting global HDAC activity or single HDAC enzymes decreased lung fibrosis and ECM deposition in bleomycin-treated mice [164,168,169,170,171]. Of note, HDAC inhibitors have been known for a long time as successful anticancer agents as they specifically induce cell cycle arrest and apoptosis in “abnormal” cancer cells, whereas normal healthy cells are relatively resistant to HDAC-inhibitor-induced cell death [172,173]. Moreover, the abovementioned preclinical studies in lung fibrosis reveal that HDAC inhibitors may also offer a new therapeutic strategy in IPF by blocking fibrotic remodelling through (i) suppression of profibrotic gene expression, (ii) restoration of antifibrotic genes and (iii) increasing myofibroblast susceptibility to apoptosis.

Importantly, similar to myofibroblasts, the very same Class-I and Class-II HDACs were also found to be upregulated in abnormal “proliferative” KRT5^+^ bronchiolar basal cells covering fibroblast foci, distal airspaces and honeycomb cysts in IPF lungs, whereas proSP-C^+^ AECII revealed a marked lack of many HDAC enzymes, suggesting that HDACs may govern the aberrant bronchiolization process of distal alveoli in IPF as well [165,174]. In agreement, Prasse et al. (2019) observed that airway/bronchiolar basal cells are increased in bronchoalveolar lavage (BAL) fluids of IPF patients and that this signature was associated with a lower survival rate [175]. Moreover, bronchial epithelium in IPF has been recognised to contribute to fibrogenesis, as it indicates the abnormal production of profibrotic growth factors, especially insulin growth factor-1 (IGF-1), TGF-β and PDGF [10].

Taken together, we suggest that in IPF, repetitive AEC injury in a (genetically susceptible) ageing individual leads to abnormal HDAC overexpression and HDAC-mediated epigenetic reprogramming in lung fibroblasts as well as bronchiolar basal cells, resulting in excessive production of profibrotic mediators, persistent AECII injury, progressive bronchiolisation, and the ongoing activation and persistence of lung fibroblasts/myofibroblasts. We thus believe that HDACs offer novel molecular targets for IPF therapy, and HDAC inhibitors may be promising therapeutic agents for the treatment of IPF. In the following chapters, the different HDAC classes, their inhibitors, and the role of HDAC enzymes in the pathogenesis of pulmonary fibrosis are described.

### 1.4. The HDAC Family/HDAC Classes and Their Function—Lessons from Cancer Research

The HDAC enzymes can be grouped into four distinct groups based on function, DNA sequence and domain organisation, and, to date, there are 18 members [176]. Class I HDACs comprise HDAC1, -2, -3 and -8, and these are widely expressed and are found mainly in the nucleus of cells. Class II HDACs are subdivided according to the presence of one or two catalytical domains. HDAC4, -5, -7 and -9 harbour one catalytically active site and are grouped into Class IIA in contrast to Class IIB, comprising HDAC6 and -10, containing two catalytic domains. In contrast to Class I HDACs, the Class IIA HDACs show a more restricted pattern of expression and are located mainly in the cell cytoplasm but can shuttle into the nucleus in a signal-dependent manner, indicating that they are unique signal transducers able to transduce signals from the cytoplasm to chromatin in the nucleus. Once inside the nucleus, Class IIA HDACs interact with myocyte enhancer factor 2 (MEF2) and other transcription factors, mainly acting as transcriptional corepressors. The Class IIB member HDAC6 is mainly cytoplasmic, and HDAC10 is pancellular. Class IV contains only one member, HDAC11, which is found in the nucleus and shares homology with Class I as well as Class II HDACs [176]. Whereas Class I, II and IV HDACs require zinc dications (Zn^2+^) for catalysis, the Class III deacetylases, the sirtuins 1–7 (*SIRT1–7*), use oxidised nicotinamide adenine dinucleotide (NAD^+^) as a co-factor [176,177].

The first identified substrates of HDACs were the histones. HDACs deacetylate the ε-amino group of lysines located at the N-terminal tail of histones, which leads through chromatin compaction to a repressive chromatin formation (heterochromatin) and the suppression of gene expression [166,176,177]. In contrast, histone acetyltransferases (HATs), such as p300, counteract histone deacetylation through the introduction of acetyl groups from acyl-CoA to histone N-terminal tails, which generates an open and relaxed chromatin structure (euchromatin), enabling transcription factors to access DNA and to activate their target genes [178]. In this regard, acetylated histones also serve as binding sites for bromodomain-containing proteins (BRDPs) to recruit macromolecular transcriptional complexes at gene promoter regions [179]. Therefore, HATs generally have the opposite function to HDACs, and the balance between the actions of these enzyme families serves as a critical regulatory mechanism for gene expression.

Further, a continuously growing number of non-histone substrates of HDACs and HATS have been described [180,181]. Many of these proteins are signal transducers (e.g., SMADs) or transcription factors (TFs), such as p53, Sp-1, AP-1, erythroid differentiation factor GATA1, runt-related transcription factors (RUNXs)**,** nuclear factor NF-kappa-B (NF-κB) and STATs (e.g., STAT3), and, therefore, changes in the transcriptome/cellular signalling due to altered acetylation status of such TFs as a result of imbalanced HDAC/HAT activity can be the consequence of direct modulation of TF activities. For example, HDAC1 and HDAC2 catalyze the deacetylation of lysine residues (K320, K373, K382] of the tumour suppressor p53, resulting in the impaired DNA binding of p53 and the inhibition of its proapoptotic transcriptional activity, such as the activation of *CIP1* (p21 or cyclin-dependent kinase inhibitor 1), *PIG3* (=*TP53I3*, p53-induced gene 3) and the caspase 9-activating gene *NOXA* (=*PMAIP1*, phorbol-12-myristate-13-acetate-induced protein 1) [182,183]. “Non-TF substrates” of HDACs are peroxiredoxin (PRDX)1 and 2, β-catenin, heat shock protein 90 (HSP90), cortactin, and α-tubulin, which are all deacetylated by the cytoplasmic and cytoskeleton-associated HDAC6 [184,185,186,187]. The deacetylation of HSP90 by HDAC6 has been associated with the stabilisation of several HSP90 oncogenic client proteins, such as AKT, hypoxia-inducible factor 1-alpha (HIF1-α), surviving and TERT, and is thus linked to carcinogenesis [186,188]. In addition, HDAC6 plays a vital role in the proteolysis pathway of misfolded proteins as it deacetylates α-tubulin for induction of aggresome formation, in which (cytotoxic) ubiquitylated protein aggregates are sequestered for lysosomal degradation and autophagic clearance, thereby attenuating ER stress [187]. HDAC6-dependent autophagy is considered to confer malignancy and aggressiveness to cancer cells and is linked to resistance to proteasome inhibitors (e.g., bortezomib) in patients with various cancers [189]. In addition, HDAC6 is implicated in metastasis formation through the induction of TGF-β-dependent epithelial–mesenchymal transition (EMT) [190,191]. HDAC6 is also involved in anti-apoptosis by deacetylating the Ku70 protein, which then forms a complex with BAX, a proapoptotic protein, allowing the inhibition of apoptosis [192]. In aggregate, overexpression of HDAC6 gives rise to cancer.

Importantly, in the vast majority, upregulation of HDAC enzymes is associated with cell proliferation, migration, cell growth, transformation and anti-apoptosis. Thus, HDAC overexpression is a common and salient feature of various cancers [166,193,194]. The Class I HDACs, especially HDAC2, epigenetically silence pro-apoptotic genes *CIP1*/p21, *PUMA* (p53 upregulated modulator of apoptosis), APAF1 (apoptotic protease-activating factor 1) and *GADD45A*/*DDIT1* (growth arrest and DNA damage-inducible protein GADD45 alpha) [182,195,196]. In addition, Class I HDACs contribute to adenocarcinoma metastasis through the induction of EMT via loss of E-cadherin expression due to epigenetic silencing by a transcriptional repressor complex containing the TF Snail (zinc finger protein SNAI1) acting in concert with HDAC1 and HDAC2 [197]. Moreover, HDAC2 supports its own expression in cancers via repressing the degradation of its transcription factor β-catenin and also promotes the expression of the proto-oncogene c-Myc [182]. Overexpression of HDAC3 is also correlated with poor prognosis in various cancers as it participates in *CIP1*/p21 repression together with HDAC1 and -2 through histone deacetylation at the proximal *CIP1* promoter or via p53 deacetylation and inhibition [198,199]. Similarly, Class IIA HDACs also promote tumorigenesis: HDAC4 promotes the growth of colon cancer cells or the progression of epithelial ovarian cancer via epigenetic *CIP1*/p21 repression by acting as a corepressor in a complex with HDAC3 [200,201] and mediates cisplatin resistance in various cancer cells through the dysregulation of autophagy and apoptosis pathways [202,203]. HDAC5 promotes the migration and invasion of hepatocellular carcinoma via increasing the transcription of hypoxia-inducible factor-1α (HIF1-α) under hypoxic conditions [204]. Further, HDAC5 displays a significant upregulation in lung cancer and increases the proliferation and invasion of lung cancer cells through the upregulation of DLL4 (Delta-like protein 4), Notch-1 (Neurogenic locus notch homolog protein 1) and Twist-1 (Twist-related protein 1) [205]. HDAC7 has been shown to protect from apoptosis by inhibiting c-Jun expression [206] and contributes to carcinogenesis by transcriptional activation of c-Myc [207]. HDAC7 also inhibits the expression of the tumour suppressor gene *JUP* (Junction plakoglobin) to promote lung cancer cell growth and metastasis [208]. Further, HDAC7 levels are increased in RAS-transformed cells, in which this protein favours the proliferation and growth of cancer stem-like cells and the invasive features of such cells [209].

Of note, HDAC1, -2, -3, -4, -5, -6 and -7 appear to be most crucial for the proliferation, aggressiveness and apoptosis resistance of cancer cells, and high expression levels of these HDACs in tumours are associated with a poor prognosis for the cancer patients [182,192,204,210,211,212]. In contrast, the depletion of many HDACs is associated with growth arrest and apoptosis. Targeted disruption of both *Hdac1* alleles in mice has been shown to result in embryonic lethality due to severe proliferation defects and retardation in development, which was correlated with increased expression of cyclin-dependent kinase inhibitors p21*^Cip1^* and p27*^Kip1^* [213]. Regarding the loss of HDAC2, there are contrasting reports in the literature. One study found that homozygous *Hdac2*^(−/−)^ mice died within the first 24 h after birth from severe cardiac malformations [214], whereas another reported that mice harbouring a *lacZ* insertion in *Hdac2* (gene-trap method) indicated partial perinatal lethality, and surviving mice with null mutation were generally indistinguishable from wild-type mice by 2 months of age [215].

The phenotypes of other (germline) full knockout of HDAC enzymes *Hdac3*, *Hdac4*, *Hdac7* and *Hdac8* are lethal, whereas *Hdac5*^(−/−)^ and *Hdac*9^(−/−)^ knockout mice are viable but with cardiac defects [177]. *Hdac6*^(−/−)^ and *Hdac11*^(−/−)^ mice are viable with no obvious phenotype [216,217]. Although α-tubulin is dramatically hyperacetylated in multiple tissues of *Hdac6*^(−/−)^ mice, the normal phenotype of these mice indicates that this is not detrimental to normal development [216]. The knockout phenotype of *Hdac10* is yet not determined, and little is known about the functions of HDAC10 [177].

The Class III HDACs are represented by the mammalian sirtuin protein family and comprise seven members (sirtuins1–7, *SIRT1–7*) of HDAC enzymes that differ in subcellular localisation and enzymatic activity and which require NAD^+^ for their catalytic activity. Gene products of *SIRT1* and *SIRT2* are found in the nucleus and cytoplasm, whereas *SIRT6* and *SIRT7* encode nuclear proteins, while gene products of *SIRT3*, *SIRT4* and *SIRT5* are localised in mitochondria [218]. The dependence of sirtuins on NAD^+^ links their enzymatic activity directly to the energy status of the cell, such as the cellular NAD^+^:NADH ratio and the absolute levels of nicotinamide, which is generated through NAD^+^ hydrolysis during lysine deacetylation and is an inhibitor of sirtuin activity itself [219]. Sirtuins are best known for their role in ageing and have been shown to prolong the mean and maximal life spans in many species across all taxonomic groups (yeast, worms, flies, mice, primates) in response to caloric restriction or activation with molecules, such as resveratrol, a potent inducer of *SIRT1*/*Sirt1* expression [218,220,221].

Because sirtuins are structurally and mechanistically distinct from Classes I, IIA, IIB and IV of histone deacetylases and are not inhibited by the widely used Zn^2+^-dependent HDAC inhibitors, they will not be covered in this review.

### 1.5. Histone Deacetylase Inhibitors

Several natural and synthetic compounds are currently known to inhibit HDACs. Since HDAC inhibitors do not inhibit all HDAC enzymes to the same extent, these agents can be grouped into pan-, Class II- and Class I-specific inhibitors [222]. Hydroxamic acids, for example, TSA (trichostatin A, which occurs naturally), belinostat, SAHA (suberoylanilide hydroxamic acid, also known as vorinostat) and LBH589 (panobinostat) are pan-HDAC inhibitors targeting (all) Class I, Class II and Class IV HDACs [222]. The related, hydroxamic acid-based pan-HDAC-inhibitor SB939 (pracrinostat) selectively inhibits Class I, II, IV HDACs except HDAC6 [223]. In contrast, the short-chain fatty acid valproic acid (VPA), the benzamide MS-275 (entinostat), and the bicyclic tetrapeptides FK228 (romidepsin) and spiruchostatin A are rather Class I-specific HDAC inhibitors [222]. The short-chain fatty acids sodium butyrate (natural compound) and 4-phenyl-butyrate (4-PBA, synthetic compound) have been reported to inhibit Class I and Class IIA HDACs (but not Class IIB HDACs) in relatively high, millimolar working concentrations [224]. The synthetic compounds TMP269, MC1575 and MC1568 have been described as specific inhibitors for Class IIA HDACs [225,226].

Importantly, the above-listed Class I and pan-HDAC inhibitors have been reported as successful anticancer agents as they induce cell-cycle arrest, differentiation and/or apoptosis in cancer cells by increasing the acetylation status of the chromatin and various non-histone proteins, such as p53, leading to their stabilization and activation. Moreover, it has been reported that both HDAC inhibitor types possess the ability to selectively induce apoptosis in “abnormal” tumour cells, whereas normal cells are relatively resistant to HDAC-inhibitor-induced cell death [172,173]. In contrast, much less is yet clear about the effects of Class IIA HDAC inhibitors in cancer cells, which still require further investigation in preclinical studies.

Importantly, HDAC inhibitors SAHA, pracrinostat, belinostat and romidepsin are FDA-approved drugs for some T-cell lymphomas [222,223,227,228], with panobinostat for multiple myeloma [229]. Pracrinostat has also been approved in 2014 as an orphan drug for acute myelocytic leukaemia (AML) [223]. Valproic acid has been in medical use since 1962 for the treatment of epilepsy and bipolar disorder, and it is marketed under the brand names Depakene and Epival (both Abbott Laboratories) [230]. It has also been tested in clinical trials as an anticancer agent and has demonstrated a promising clinical response [231]. 4-PBA, though not in oncology, was approved by the FDA in 1996 for the treatment of urea cycle disorders [224] and is now being investigated for therapy in some types of cancer [232]. In addition, a new clinical-stage HDAC inhibitor, CG-745, specific for Class I HDACs and Class IIB HDAC6, has recently been granted an orphan drug designation by the FDA for the treatment of patients with pancreatic cancer [233]. All these encouraging results justify that more and more HDAC inhibitors are currently being investigated in a number of clinical trials as part of mono- or combination therapies for the treatment of various cancers. Class I, II, IV and pan-HDAC inhibitors chelate the Zn^2+^ cation within the enzyme active site, resulting in the inhibition of HDAC activity. Importantly, the Class III HDACs sirtuins1–7, which contain a NAD^+^-dependent catalytic domain, are insensitive to these agents [172,177,222].

Due to the broad activity of classical Class I and pan-HDAC inhibitors across HDAC isoforms, the development of second-generation HDAC inhibitors has been focused on improving the selectivity of HDAC inhibitors, resulting in the discovery of a series of isoform-specific HDAC inhibitors. Until now, most of the agents developed and reported in existing articles have selectivity for HDAC3 [234], HDAC6 [174,189,235,236] and HDAC8 [171,237] and are currently being evaluated in preclinical studies. At present, only the HDAC6 inhibitor ACY-1215 (Ricolinostat) has been tested in a phase 2 clinical trial as a treatment strategy for relapsed/refractory lymphoid malignancies (NCT02091063) [238].

In contrast to Class I, II, and IV HDAC inhibitors, much less is known about sirtuin inhibitors. The protein-deacetylating activities of both sirtuin-1 and sirtuin-2 can be inhibited simultaneously by the small molecules tenovin-1/-6 or sirtinol (with no effects on other sirtuins and Zn^2+^-dependent HDACs), which are currently being targeted as potential therapeutic agents for cancer since they induce p53-dependent proapoptotic activity in malignant cells while having no effect on normal cells [239,240]. The indole compound selisistat (EX527) is a potent and selective sirtuin-1 inhibitor [240]. Surprisingly, until now, no clinical trials are underway to evaluate the efficacy of Class III HDAC inhibitors in cancer. Because the Zn^2+^-dependent HDACs (Classes I, IIA, IIB and IV) are recognised as “classical HDACs” and common targets for therapy, the Class III sirtuins and their inhibitors are not in the scope of this review.

## 2. Imbalanced Histone Deacetylase (HDAC) Activities in Idiopathic Pulmonary Fibrosis: Effects and Therapeutic Correction

Increased activity and overexpression of histone deacetylases (HDACs) have been described for a long time in various pathological conditions such as cancer, cardiac hypertrophy and hypertension [166,193,241,242]. Upregulated HDAC activities are also observed in fibrotic diseases involving the heart, liver, kidneys and lungs; and experimental studies performed on animal models have shown that HDAC inhibitors can ameliorate various forms of fibrosis [164,168,169,170,243,244,245].

In IPF, where persistent fibroblast activation underlies progressive fibrotic disease, HDAC-mediated gene repression of antifibrotic molecules and proapoptotic factors appears to be a critical event [162,163,164,246]. We have recently reported that lung fibroblasts from patients with IPF exhibit a profibrotic phenotype with “cancer-like features” due to the abnormal overexpression of all Class I and Class II HDAC enzymes, which appeared responsible for their aberrant activation and persistence in IPF, presumably as the result of changes in expression profiles and cellular signalling due to alterations in the acetylation status of the chromatin and various non-histone proteins [165]. In accordance, it could be demonstrated that the pan-HDAC inhibitor panobinostat (LBH589), an FDA-approved drug for the treatment of multiple myeloma since 2015 [229], reduced proliferation, collagen-I biosynthesis, and anti-apoptotic genes in IPF fibroblasts in vitro, with concomitant induction of p21^Cip1^ and ER stress-mediated apoptosis [165]. In addition, panobinostat also restored and enhanced the expression of antifibrotic genes silenced in IPF fibroblasts [162,247]. These processes were accompanied by massive chromatin and α-tubulin acetylation, confirming efficient Class I/IIA/IIB HDAC inhibition through panobinostat [165]. In addition, Jones and coworkers (2019) identified the pan-HDAC inhibitor pracrinostat, approved in 2014 as an orphan drug for AML, as a potent attenuator of lung fibroblast activation in IPF patient-derived fibroblasts [246]. Sanders and coworkers (2014) demonstrated the inactivation of IPF fibroblasts as well as the amelioration of experimental pulmonary fibrosis in response to global HDAC inhibition by SAHA [164]. The “older” pan-HDAC inhibitor SAHA was the first FDA-approved HDAC-inhibiting drug for the treatment of cancers, and it has been in clinical use since 2006 [222]. Table 1 summarises the broad therapeutic effects of various pan-HDAC inhibitors on preclinical models of lung fibrosis/IPF, which will be outlined in the following chapters of this article.

The reported evidence that alterations of protein function or chromatin accessibility through imbalanced deacetylation/acetylation are obviously key in the pathogenesis of IPF has led to the initiation of research studies to identify the exact targets and direct effects of HDAC enzymes in the setting of pulmonary fibrosis. Chromatin immunoprecipitation (ChIP) studies examining histone modifications have identified that several important anti-fibrotic genes are silenced in fibrotic and IPF fibroblasts, including *CAV1* (encoding caveolin 1) [257], *CXCL10* (encoding CXC motif chemokine 10) [247], *THY1* (encoding the anti-fibrotic receptor Thy1 membrane glycoprotein, also known as CD90) [248], *NFE2L2* (encoding the antioxidant transcription factor nuclear factor erythroid-derived 2-related factor-2, also known as NRF2) [258], *PPARG* (encoding the peroxisome proliferator-activated receptor-gamma, PPARγ) [171], *PGC1A* (encoding the PPARγ coactivator 1-alpha, PGC1-α) [246], and *COX2* (encoding cyclooxygenase-2) [162], all of which were restored upon HDAC-inhibitor treatment.

The aberrant HDAC-mediated silencing of *COX2* results in the loss of its metabolite prostaglandin E2, an autocrine anti-fibrotic mediator that controls fibroblast cellular overactivation while promoting the survival of AECII. Of note, the fibroblasts isolated from IPF patient lungs indicate reduced levels of COX2 or NRF2 even after 6 or more passages [162,258,259], hinting at the involvement of epigenetic repression mechanisms and that HDAC-mediated gene repression of antifibrotic molecules precedes growth-factor-induced profibrotic gene expression in IPF. In agreement with the increased apoptosis resistance and persistence of lung fibroblasts in IPF, proapoptotic genes *BAK* and *FAS* are also epigenetically repressed in IPF fibroblasts [163,164]. Importantly, in addition to reduced histone H3 acetylation, the promoters of *BAK* and *FAS* exhibited an increased level of trimethylated lysine 9 on histone H3 (H3K9me3), a repressive chromatin marker [163,164], indicating the close crosstalk between histone deacetylation with histone hypermethylation changes at specific histone residues [260]. In particular, histone modifications H3K9me3 and H3K27me3 are associated with decreased gene expression and linked to the increased recruitment of HDACs at promoters [260]. HDAC inhibitors have also been demonstrated to reverse repressive histone hypermethylation as ChIP assays revealed that SAHA treatment of IPF fibroblasts resulted in an enrichment of the pro-apoptotic *BAK* gene with acetylated lysine 9 on histone H3 (H3K9Ac), an active chromatin mark, and the depletion of *BAK* with H3K9Me3, thereby corresponding to increased *BAK* expression in the “corrected” IPF fibroblasts [164].

On the other side, increased expression of anti-apoptotic proteins surviving and the BCL-XL in IPF fibroblasts becomes paradoxically downregulated in response to pan-HDAC inhibitors known to increase general histone acetylation. Similarly, genes involved in myofibroblast differentiation, such as *ACTA2*, *COL1A1*, *COL3A1* and *FN,* are suppressed in primary IPF fibroblasts in response to panobinostat or SAHA [164,165,249]. Jones et al., observed that TGF-β induced-expression of *ACTA2*, *TNC*, *IL6*, *IL11* and *PDGFA* was abrogated in IPF fibroblasts upon treatment with pracrinostat [246]. In agreement with these findings, it could be demonstrated that panobinostat abrogated STAT3-phosphorylation at tyrosine 705 (Tyr705) and its fibrotic action in IPF fibroblasts, thus offering a plausible explanation for the reduction of survival- and ECM-associated genes in response to global HDAC inhibition and evidence for the involvement of HDACs in increased expression of such profibrotic genes [249]. Various reports from the cancer field suspect that Class I HDAC activity and increased deacetylation of STAT3 appear to be required for its phosphorylation at Tyr705 and nuclear translocation as HDAC1, -2 and -3 have been reported to reduce STAT3 acetylation [261,262] and as selective inhibition of Class I HDACs was sufficient in efficiently suppressing STAT3-pTyr705 phosphorylation and its signalling in a variety of malignant cells [262,263,264]. Similarly, Class I HDACs were demonstrated to be required for the activation of the extracellular signal-regulated kinase-1 (ERK/MAPK3) and PI3K pathways by TGF-β in lung fibroblasts and, thus, for the subsequent ECM gene induction dependent on these non-SMAD signalling pathways [265].

Further, Class IIA HDAC4, which is prominently upregulated in IPF versus normal fibroblasts, was shown to form a protein complex with some cytoplasmic protein phosphatases (PP) in response to TGF-β to prevent the dephosphorylation and inhibition of AKT in fibrotic lung fibroblasts for inducing and maintaining AKT-mediated profibrotic gene expression [250]. Similarly, TGF-β induced expression of ECM genes has been suspected to require Class IIB HDAC6 function in AKT activation as well, as selective HDAC6 inhibition was shown to disrupt TGF-β-elicited AKT signalling in fibrotic lung fibroblasts [235]. Together, these data suggest that HDACs also mediate profibrotic signalling in fibrotic/IPF-fibroblasts through interaction with various non-histone protein targets, which is reversed by HDAC inhibition. On the other side, it must not be excluded that HDACs mediate profibrotic gene expression due to epigenetic silencing of repressors of profibrotic genes. Importantly, temporal gene expression analyses of TGF-β-treated primary lung fibroblasts done by Jones and coworkers (2019) have revealed that TGF-β-mediated HDAC signalling repressed antifibrotic genes prior to the upregulation of profibrotic genes [246].

Interestingly, similar to IPF fibroblasts/myofibroblasts, Class I and Class II HDACs were also found to be upregulated in abnormal, “proliferating” KRT5^+^ bronchiolar basal cells at sites of aberrant re-epithelialization and co-localised with the expression of p-STAT3 and surviving [165,249]. The crucial intrinsic activity of HDACs in cell proliferation, cell migration and anti-apoptosis suggests a strong contribution of these enzymes in the “re-programming” and abnormal activation of such cells in the progressive bronchiolisation of damaged alveolar epithelium and the fibrosing process in IPF [165,174]. Further studies proved the eminent profibrotic effect of airway/bronchiolar basal cells derived from IPF patients [174,266], which, interestingly, could be largely abrogated by the selective inhibition of HDAC6 [174].

Importantly, in contrast to myofibroblasts and basal cells, expression of many HDACs appeared to be sparse or even absent in IPF-AECII undergoing proapoptotic ER stress [165], which is in agreement with the degradation and depletion of many HDAC enzymes under conditions of severe ER/oxidative stress or apoptosis [267,268,269,270,271].

**Figure 1:**
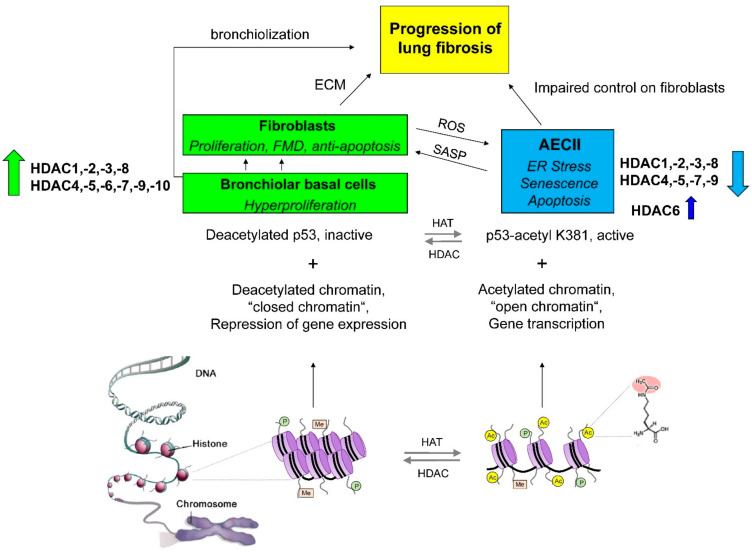
**Imbalanced histone deacetylase activities in IPF.** IPF is characterised by a significant imbalance of histone deacetylase (HDAC) activities, with an abnormal increase of HDAC expression in fibroblasts/myofibroblasts and bronchiolar basal cells, but a lack of HDAC expression in AECII due to ER stress, senescence and apoptosis. This imbalance contributes and perpetuates the fibrotic process. Abbreviations: ECM: extracellular matrix; FMD: fibroblast-to-myofibroblast differentiation; AECII: type-I/-II alveolar epithelial cell; ROS: reactive oxygen species; SASP: senescence-associated secretory phenotype; HAT: histone acetyltransferase; P = phosphorylation, Me = methylation, Ac = acetylation.

Taken together, IPF appears to be characterised by a significant imbalance of HDAC activities, with an abnormal increase of HDAC expression in fibroblasts/myofibroblasts and bronchiolar basal cells but a lack of HDAC expression in AECII due to irremediable ER stress and apoptosis. The consequent (differentially) deregulated acetylation status of histone tails and non-histone proteins/TF substrates in fibroblast populations and basal cells versus the AECII of IPF lungs may lead ultimately to altered chromatin transcription profiles and shifted cellular signalling and disturbed inter-cellular communication, which contribute to fibrosis (Figure 1). In the following chapters, the pathogenic role of different HDAC classes/HDAC isoforms in IPF and their therapeutic correction in preclinical models are described.

### 2.1. Class I Histone Deacetylases in IPF: Expression Profile, Function and Preclinical Studies

In our report from 2015, we found all Class I HDAC enzymes significantly upregulated on the proteomic level in IPF lung tissues as well as in primary IPF fibroblast isolations by immunoblotting studies, in comparison to tissues and fibroblast isolates obtained from normal lungs [165], in agreement with other studies [171]. Immunohistochemical studies confirmed strong induction and predominant localisation of HDAC1, -2, -3 and -8 in α-SMA expressing myofibroblasts of fibroblast foci, but also revealed robust expression of all Class I HDACs in abnormal, hyperplastic bronchiolar basal cells at sites of aberrant re-epithelialisation in IPF. In marked contrast, expression of Class I-HDACs was absent in the proSP-C^+^ AECII of IPF lungs [165].

In addition, ciliated bronchial cells of IPF bronchioles also indicated a prominent upregulation of HDAC2 and -3 compared to healthy control lung tissues. This observation appeared to be conceptual since KRT5^+^ expressing basal cells are the progenitors for non-ciliated Club cells and ciliated FOXJ1^+^ bronchial cells, and they are suggested to initiate the progressive bronchiolization process of alveolar spaces in IPF. Further, in agreement with various reports about expression patterns and functions of Class I enzymes, HDAC1, -2, and -3 indicated a dominant nuclear expression in the abovementioned cell types, whereas HDAC8 indicated cytoplasmic as well as nuclear localisation [165]. Further, HDAC2 and HDAC3 have been observed to be upregulated in various rodent models of pulmonary fibrosis [251,272], with predominant localisation in fibrotic lesions and lung fibroblasts [163,258,273].

Abnormal overexpression of Class I HDACs in IPF fibroblasts/myofibroblasts appears to play a crucial role in the fibrotic process, as ChIP studies by Coward and coworkers (2009) revealed that the CoREST (REST corepressor 1) and Sin3a (Sin3 histone deacetylase corepressor complex component SDS3) transcriptional corepressor complexes, which consist of HDAC1 and HDAC2, and the N-CoR (nuclear receptor corepressor 1) complex consisting of HDAC3, were bound to the promoter of the antifibrotic gene *COX2* in primary IPF fibroblasts, resulting in the deacetylation of histone H3 and H4 at the *COX2* promoter and decreased transcription factor binding, leading to diminished *COX2* expression in IPF fibroblasts [162]. Similar observations they made for the *CXCL10* gene, which is suppressed in IPF fibroblasts as well due to insufficient histone H3/H4 acetylation and repressive histone H3 hypermethylation at its promoter as a result of decreased recruitment of HATs but increased recruitment of Class I HDAC-containing transcriptional repressor complexes in IPF fibroblasts [247]. In agreement, treatment of IPF fibroblasts with the well-characterised pan-HDAC inhibitor panobinostat resulted in the restoration of *COX2* and *CXCL10* expression through the creation of an active chromatin structure at their promoters, manifested as the accumulation of acetylated histones H3 and H4 [162,247]. The malignant repression of *FAS* in fibrotic fibroblasts was also attributed in part to increased HDAC2 expression [163].

Further, profibrotic STAT3-pTyr705 phosphorylation and activation are prominently upregulated in IPF fibroblasts [73,249] and suggested to be promoted by lysine deacetylation of STAT3 through HDAC1, -2 and/or -3 [261,262]. In addition, Class I HDACs have also been reported to be involved in the activation of the upstream JAK2 kinase [264]. In agreement, STAT3-pTyr705 phosphorylation was abrogated in response to panobinostat treatment, resulting in a reduction of cell proliferation, surviving expression and ECM associated genes in IPF fibroblasts [249]. Moreover, panobinostat not only inhibited HDACs, but it also led to significant proteolysis of HDAC1 and HDAC2 and, thus, the efficient inactivation of both HDACs in IPF fibroblasts, whereas the mRNA levels for both enzymes were not affected [249]. The tumour suppressor p53 is another substrate of Class I HDACs, and the enormous loss of HDAC1/2 function in response to panobinostat was associated with the strong upregulation of p21^CIP1^ and other p53 target genes in IPF fibroblasts [165].

Interestingly, SMAD7, which is known to inhibit TGF-β/SMAD2/3 signalling, has also been identified as a non-histone protein target of HDAC1 and is destabilised through deacetylation [274]. In TGF-β-stimulated human lung fibroblasts, HDAC1 was shown to become upregulated, while SMAD7 became downregulated [252]. Further studies revealed that treatment of TGF-β-stimulated lung fibroblasts with the pan-HDAC inhibitor SAHA prevented SMAD7 deacetylation by inhibiting TGF-β-induced HDAC1 activity, resulting in increased SMAD7 expression and decreased SMAD3 phosphorylation with subsequent reduction of profibrotic signalling. SAHA also attenuated paraquat-induced lung fibrosis in rats in vivo through the restoration of Smad7 protein expression and the suppression of the canonical TGF-β pathway [252].

As can be seen, many studies characterizing the function, contribution and therapeutic correction of aberrant Class I HDAC activity in lung fibrosis are based on the use of pan-HDAC inhibitors. In the next two chapters, the therapeutic effects of specific Class I HDAC inhibitors and Class I isoform-selective inhibitors in preclinical models of lung fibrosis are summarised.

#### 2.1.1. Class I HDAC Inhibitors in Preclinical Studies of Lung Fibrosis

The crucial role of Class I HDACs in lung fibrosis is also underscored by the fact that their specific inhibition through spiruchostatin A has been shown to significantly reduce fibroblast proliferation and myofibroblast markers on protein level in TGF-β-stimulated IPF fibroblasts in vitro. Spiruchostatin A also increased histone H3 acetylation and *CIP1*/p21 expression, suggesting that direct cell-cycle regulation was the mechanism for inhibiting proliferation [275]. The related FDA-approved Class I inhibitor romidepsin indicated the very same effects, in addition to its profound capability in downregulating the protein expression of lysyl oxidase (LOX), an enzyme involved in collagen-crosslinking, in TGF-β-treated IPF fibroblasts. Interestingly, it was also demonstrated that romidepsin exerted minimal effects on primary normal human AECII at doses that markedly suppressed the proliferation of IPF fibroblasts [169]. In vivo, romidepsin inhibited bleomycin-induced lung fibrosis in mice in association with suppression of LOX expression [169].

Interestingly, the weak Class I inhibitor valproic acid (VPA) was also shown to efficiently reduce cell proliferation, surviving expression, collagen-I protein turnover in IPF fibroblasts, and these effects were accompanied by the significant degradation and loss of HDAC2 [165], an effect which has been observed in various cells upon VPA treatment [276]. Although VPA has been shown to selectively inhibit STAT3-pTyr705 phosphorylation in various malignant and non-malignant cells [263,277], this effect, however, has yet not been addressed in fibrotic fibroblasts. In fibrotic A549 cells, VPA was shown to decrease TGF-β induced histone deacetylation and subsequently restored TGF-β downregulated epithelial genes in A549 cells, but only partially inhibited EMT, as many profibrotic genes upregulated by TGF-β were not suppressed by VPA, with the exception of *COL1A1* [278]. Similarly, in IPF fibroblasts, VPA significantly suppressed pro-collagen-I expression but upregulated the α-SMA level [165]. Interestingly, silencing of *HDAC2* by RNA interference (RNAi) was also shown to further increase *ACTA2* expression in TGF-β-stimulated human skin fibroblasts, whereas *HDAC8* silencing significantly suppressed TGF-β-induced *ACTA2* expression in these cells [253]. Depletion of *Hdac3* by RNAi resulted in the downregulation of TGF-β induced disintegrin and metalloproteinase domain-containing protein-12 (*Adam12*) and metalloproteinase inhibitor-1 (*Timp1*) expression in mouse fibroblasts but was strictly dependent on the suppression of TGF-β-activated ERK (MAPK3) and PI3K signalling pathways and, thus, restricted to ECM genes dependent on these Smad-independent signalling pathways as TGF-β-upregulated *Pai1* expression was unaffected [265].

Interestingly, in contrast to in vitro studies, VPA was shown to significantly attenuate EMT and lung fibrosis in bleomycin-treated mice in vivo, which was associated with Smad2/3 deactivation but without Akt cellular signal involvement [170]. In bleomycin-treated rats, it reduced oxidative stress and proinflammatory cytokines in injured lungs, together with significant improvement of the histopathological picture [254]. The failure of VPA to deactivate Akt signalling is presumably due to the fact that VPA specifically inhibits the activities of HDAC1 and HDAC2 [165,222] but not of HDAC3 (involved in mediating TGF-β-induced PI3K signalling [265], as mentioned above). In agreement, the Class I HDAC inhibitor entinostat (MS-275), specific for HDAC1 and HDAC3, also led to the inactivation of the PI3K/Akt pathway in TGF-β-stimulated lung fibroblasts [265]. In addition, entinostat was shown to suppress TGF-β-induced expression of SPARC, a matricellular protein involved in the ECM turnover and apoptosis resistance of myofibroblasts in cultured human lung fibroblasts. In detail, entinostat restored the expression of ARHGEF3 (Rho guanine nucleotide exchange factor 3, also known as XPLN = exchange factor found in platelets and leukemic and neuronal tissues), a negative regulator of *SPARC* expression, as *ARHGEF3* was repressed by TGF-β-induced HDAC1/3 signalling [279]. In summary, all these studies prove the significant antifibrotic efficacy of Class I-selective HDAC inhibitors in preclinical models of lung fibrosis and underscore the crucial role of Class I HDACs in mediating profibrotic signalling in IPF/fibrotic lung fibroblasts.

However, one contrary report also exists by Rubio and coworkers, describing reduced HDAC activity in nuclear extracts of IPF versus normal fibroblasts, despite evidenced upregulation of HDAC1 and HDAC2 in IPF fibroblasts [280]. Hence, HDAC activity was upregulated in the cytosolic fraction of IPF fibroblasts. Subsequent studies revealed that histone acetyltransferase p300 inactivated the nuclear activity of HDAC1 by acetylation, thereby resulting in the disruption of the multicomponent RNA-protein complex “MiCEE”, which is considered a repressor of general gene transcription in IPF fibroblasts. Indeed, this could explain the exaggerated expression of ECM genes during fibrogenesis in IPF fibroblasts. In line with this suggestion, inhibition of p300 resulted in reduced ECM production in IPF fibroblasts in vitro and decreased lung fibrosis in bleomycin-treated mice in vivo [280]. Taken together, the results from this very interesting study support a key role of active p300 and Class I HDAC inactivation during IPF and propose p300 inhibition as therapy for IPF, but stands in vast contrast to numerous reports suggesting HDAC inhibitors for targeting increased Class I HDAC activity in fibrotic fibroblasts as a therapeutic option for IPF patients.

The effects of Class I HDAC inhibitors on bronchiolar basal cells and the aberrant bronchiolization process in IPF remain to be determined, which would help to clarify the role of the eminent upregulation of all Class I HDACs in basal cells, Clara cells as well as FOXJ1 expressing the bronchial cells of IPF lungs. Interestingly, COX2 protein expression was also previously observed to be diminished in the bronchiolar epithelial cells of IPF lungs [281], which might be caused by aberrant Class I HDAC-mediated epigenetic repression of *COX2* expression, as observed in IPF fibroblasts.

The effects of Class I HDAC inhibitors and Class I isoform-selective inhibitors on preclinical models of lung fibrosis/IPF are summarised in Table 2.

#### 2.1.2. Class I Isoform-Selective Inhibitors in Preclinical Models of Lung Fibrosis

Recently, published work highlights a crucial role of single Class I HDAC enzymes, in particular HDAC3, in progressive fibrotic lung diseases as the HDAC3-selective inhibitor RGFP966 effectively reduced pulmonary fibrosis in bleomycin-treated wild-type mice and conditional *Hdac3*^(−/−)^ mice were largely resistant to bleomycin-induced lung fibrosis [258]. HDAC3 was observed to be overexpressed in IPF lungs and to be preferentially upregulated in the fibrotic phase in bleomycin-injured lungs at day 14 and day 21 post-bleomycin and was found to repress *Nrf2* expression in concert with the profibrotic Forkhead box M1 (FOXM1) transcription factor. Conversely, RGFP966 treatment reduced the HDAC3 and FOXM1 bindings and elevated histone H3 acetylation levels at the *Nrf2* promoter, resulting in *Nrf2* derepression and increased Nrf2-induced expression of antioxidants. In addition, this treatment also restored epithelial E-cadherin expression while downregulating profibrotic ECM molecules [258]. Similarly, in IPF, NRF2 is largely absent in myofibroblasts within fibroblast foci [282], while increased HDAC3 expression in these foci is observed [165]. Taken together, aberrant overexpression of HDAC3 and its exerted suppression of NRF2 plays a pivotal role in lung fibrosis, including IPF, which can be attenuated by selective inhibition of HDAC3.

Moreover, HDAC3 has also been reported to negatively regulate miR-19a-3p to increase interleukin 17 receptor A (IL17RA) expression in rheumatoid arthritis (RA)-associated interstitial lung disease (ILD) [273]. Interestingly, both HDAC3 and IL17RA were found to be much more upregulated in the lung tissues of RA-ILD patients than in patients with IPF versus healthy controls. Further analysis revealed a positive correlation of expression between HDAC3 and IL17RA in RA-ILD. Using an RA-ILD mouse model, HDAC3 downregulated miR-19a-3p expression in the lung fibroblasts of these mice, which resulted in increased IL17/IL17RA signalling and ECM protein expression, an effect that was abrogated by overexpression of miR-19a-3p or siRNA-mediated *Il17ra* or *Hdac3* silencing. Of note, delivery of *Hdac3*-targeting siRNA to RA-ILD mice attenuated lung fibrosis in mice in vivo [273].

Similar to the effects of HDAC3 inhibitor, selective inhibition of Hdac8 was also shown to ameliorate bleomycin-induced lung fibrosis in mice through the reduction of ECM synthesis [171]. Interestingly, HDAC8 has been regarded for a long time as a marker for myofibroblasts and smooth muscle cells, where it displays a strong association with α-SMA, and *si*RNA-mediated silencing of *HDAC8* revealed that it is essential for smooth muscle cell contractility [283,284]. HDAC8 expression has been demonstrated to increase in human lung fibroblasts in response to TGF-β, where it deacetylated chromosomes protein-3 (SMC3), a specific substrate of HDAC8, but not of other HDACs in the nucleus, while inducing α-SMA stress fibre formation in the cytoplasm of TGF-β-treated fibroblasts. This process was associated with increased production of ECM proteins and was abrogated by *HDAC8* silencing or inhibition of HDAC8 with NCC170. Further studies revealed that HDAC8 inhibited antifibrotic PPARγ-signalling through the repression of the *PPARG* transcription as HDAC8 was found in ChIP assays to deacetylate histone H3 at *PPARG* gene enhancer regions. *Vice versa*, HDAC8 inhibition ameliorated the TGF-β-induced loss of the active chromatin marker H3K27ac and restored *PPARG* expression. Interestingly, HDAC8 inhibition did not appear to alter the TGF-β-induced phosphorylation of SMAD2, SMAD3 or AKT, suggesting that PPARγ upregulation was at least partially responsible for the suppressive effects of HDAC8 inhibition on TGF-β induced fibroblast-to-myofibroblast transformation [171].

Taken together, these three studies involving animal models of lung fibrosis highlight the obviously potent antifibrotic efficacy of pharmacological inhibition or genetic knockdown of single Class I isoforms to block the evolvement of lung fibrosis (Table 2) and emphasise an underestimated dominant role of HDAC3 and HDAC8 in the pathogenesis of fibrotic lung disease.

#### 2.1.3. Loss of Sin3-HDAC1/HDAC2 Repressor Complex Activity in AECII Results in Alveolar Senescence

The observed eminent lack of Class I HDACs in proSP-C+ expressing AECII in IPF is not only plausible due to increased alveolar ER stress and apoptosis but also considerably mirrored by increased p53-p21CIP1 activation and senescence in IPF-AECII, which has been widely reported by many scientists [17,118,119,120]. AECII senescence in IPF has been associated with p21-induced cell-cycle arrest and gradual apoptotic cell loss and with the senescence-associated secretory phenotype (SASP) contributing to myofibroblast expansion [17,120]. In addition, ER stress involving Chop has recently been shown to promote AECII senescence and SASP phenotype in two different experimental models of pulmonary fibrosis [285,286].

Moreover, it could be recently demonstrated that the AECII-specific conditional loss of *Sin3a*, a key component of the Sin3-HDAC1/HDAC2 corepressor complex, results in profound AECII senescence and spontaneous progressive lung fibrosis in mutant mice that closely resembles the pathological remodelling seen in IPF [120]. Fibrosis in these mice was diminished either by the selective loss of p53 function in AECII or by the ablation of senescent AEC cells through systemic delivery of senolytic drugs [120]. Previous research by the same group showed that loss of *Sin3a* in mouse early foregut endotherm led to a specific and profound defect in lung development, with complete loss of epithelial cells at later stages, resulting in the death of neonatal pubs at birth due to respiratory insufficiency. Further analyses revealed that embryonic lung epithelial cells adopted a senescence-like state with permanent cell-cycle arrest in the G1 phase before their demise [287]. Similarly, mice with foregut endoderm-specific, global deletion of *Hdac1* and *Hdac2*, but not individual loss of these HDACs, died at birth because of respiratory distress due to loss of Sox2 expression and a block in proximal airway development [288]. Total loss of *Hdac3* in the developing lung epithelium led to the diminished spreading of AECI cells and a disruption of lung sacculation, and newborn mutant mice died shortly after birth [289]. In adult mice, conditional loss of *Hdac1*/*Hdac2* in the proximal airway epithelium led to increased expression of the cell-cycle regulators Rb1 (retinoblastoma-associated protein), p21^Cip1^, and p16^Ink4a^ (cyclin-dependent kinase inhibitor 2A), resulting in a loss of cell-cycle progression and defective regeneration of Sox2-expressing airway epithelium after naphthalene injury [288].

In summary, all these data suggest that AECII senescence and apoptosis in IPF are mediated by both ER stress and a lack of Class I HDACs.

### 2.2. Class IIA Histone Deacetylases in IPF: Expression Profile, Function, and Preclinical Studies

In our report from 2015, we also found all Class IIA HDACs (HDAC4,-5,-7 and -9) significantly upregulated on the proteomic level in IPF lung tissues in comparison to normal lungs. Immunohistochemical analyses showed strong overexpression of all Class IIA enzymes in myofibroblasts within fibroblast foci and abnormal hyperplastic bronchiolar epithelium, including ciliated bronchial cells of IPF lungs. Immunoblot analyses revealed, especially for HDAC4 and HDAC7, a striking upregulation in IPF versus control fibroblasts, which was also evident on the mRNA level in addition to robust *HDAC5* and (Class IV) *HDAC11* upregulation. Further, all Class IIA HDACs indicated a predominant cytoplasmic localisation in myofibroblasts in IPF [165,290], which appeared to be necessary for their profibrotic function. Co-immunoprecipitation studies indicated that HDAC4 interacts directly with α-SMA and appears to be required for a-SMA fibre formation and cell contraction in lung fibroblasts in response to TGF-β stimulation [290]. Further, the translocation of Class IIA HDAC enzymes (HDAC4,-5,-7 and -9) from the nucleus to the cytoplasm is associated with the activation of transcription factors myocyte-enhancer factor-2 (MEF2) and serum response factor (SRF), which activate myogenic genes such as *ACTA2* and which are repressed by nuclear localisation of Class IIA HDACs. The HDAC-mediated repression of MEF2 activity is abrogated by calcium/calmodulin-dependent protein kinase (CaMK) through phosphorylation of HDAC4,-5,-7,-9 “in response to stress stimuli”, resulting in consequent dissociation from MEF2 and their nuclear export, enabling MEF2 to stimulate profibrotic and progrowth genes [291,292,293].

Gene silencing studies using RNAi technology identified HDAC4 as an HDAC that mediates TGF-β-induced differentiation of normal lung/skin fibroblasts into myofibroblasts [250,253]. Additionally, here, the cytoplasmic localisation of HDAC4 was required for the activation of the AKT signalling pathway, which is necessary for the expression of *ACTA2* and other ECM genes in response to TGF-β [250]. As a mechanism, it has been suggested that HDAC4 captures cytoplasmic serine/threonine phosphatases PP1 and PP2A, thereby protecting AKT from being dephosphorylated. Conversely, RNAi-mediated silencing of *HDAC4* as well as pan-HDAC inhibition through TSA led to the disruption of the HDAC4/PP1/PP2A complex and the liberation of PP1 and PP2A, followed by the dephosphorylation and inactivation of AKT, with the consequent blockade of TGF-β-stimulated α-SMA expression [250]. Importantly, abrogation of TGF-β-induced fibroblast-to-myofibroblast transformation by TSA was SMAD2/3-independent [250]. It was also suggested that TGF-β promoted HDAC4 nucleus-to-cytoplasm translocation through increased NOX4-derived ROS production, which leads to cysteine oxidation and intramolecular disulfide bond formation within HDAC4, facilitating its nuclear export and consequent profibrotic effects in the cytoplasm of stimulated lung fibroblasts [290]. Taken together, Class IIA HDAC enzymes such as HDAC4 alter gene expression profiles and cellular signalling not only through histone deacetylation but also through protein–protein interactions in the cytoplasm.

Interestingly, HDAC4 revealed an entirely cytoplasmic expression in KRT5 expressing basal cells of hyperplastic IPF bronchioles or adjacent to fibroblast foci, whereas luminal ciliated bronchial cells in IPF bronchiolar structures indicated a dominant nuclear localisation of HDAC4 [165]. Nuclear functions of HDAC4 include the repression of MEF2 activity and/or p21^CIP1^ expression. ChIP analyses demonstrated that HDAC4 represses p21*^CIP1^* expression as a component of the HDAC4–HDAC3–N–CoR corepressor complex bound to the proximal *CIP1* promoter [200,201]. HDAC4 was also suggested to be involved in the repression of *FAS* in fibrotic fibroblasts, together with Class I HDAC2 [163]. In IPF fibroblasts, HDAC4 was observed in both the cytoplasm and the nucleus. In aggregate, HDAC4 appears to manifest its distribution in the cytoplasm and/or nucleus depending on cell type, the stage of cell differentiation, and the physiological condition.

More recently, Class IIA HDAC7 has been identified as a key enzyme facilitating TGF-β-mediated regulation of key pro- and anti-fibrotic genes in IPF. Specific HDAC gene silencing (*HDAC1* through *HDAC11*, except *HDAC6*) by RNAi in TGF-β-stimulated IPF fibroblasts with the use of *ACTA2* transcript levels as a readout parameter revealed that the silencing of *HDAC7* by RNAi was the most effective in reducing TGF-β-induced *ACTA2* expression in primary IPF fibroblasts [246]. Aside from *ACTA2*, the knockdown of *HDAC7* by RNAi resulted in a significant reduction of TGF-β-upregulated profibrotic mediators *NOX4* and *CTGF* in stimulated IPF fibroblasts, while expression of the TGF-β-suppressed antifibrotic gene *PGC1A* was increased. Moreover, silencing of *HDAC7* also led to reduced expression of *HDAC2*, *HDAC6*, *HDAC8* and *HDAC10* but increased *HDAC9* expression, suggesting that HDAC7 affected gene expression profiles through the regulation of the expression of other HDACs in response to TGF-β [246]. Interestingly, RNAi-mediated knockdown of *HDAC7* was also shown to reduce TGF-β induced SMAD2/3 activation, myofibroblast differentiation and ECM production in primary fibroblasts derived from human Peyronie’s disease plaque [294]. In TGF-β-treated skin fibroblasts from patients with systemic sclerosis, *HDAC7* silencing resulted in reduced collagen-I and collagen-III production [295]. A recent study showed that HDAC7 was involved in endothelin-1-induced production of CTGF in lung fibroblasts through the formation of a transcriptional complex with p300 and AP-1 and recruitment to the *CTGF* promoter region, resulting in *CTGF* expression. In detail, endothelin-1 promoted HDAC7 translocation from the cytosol to nucleus and HDAC7 initiated AP-1 transcriptional activity (surprisingly) through the recruitment of p300 and consequent p300-mediated AP-1 acetylation. Conversely, RNAi-mediated silencing of either *HDAC7* or *EP300* suppressed endothelin-1-induced *CTGF* expression [296]. Taken together, these results indicate a crucial role for *HDAC7* in cytokine/growth factor-induced expression of profibrotic molecules during fibrogenesis. Interestingly, pan-HDAC inhibitors such as TSA [295] and panobinostat [165] were observed to result in the profound suppression of HDAC7 on the transcriptomic and proteomic levels in primary IPF fibroblasts. Even the weak Class I inhibitor VPA reduced it significantly on mRNA and protein levels [165].

In contrast to HDAC4 and HDAC7, much less is known about the role and functions of HDAC5 and HDAC9 in lung fibrosis. Interestingly, silencing of *HDAC5* by RNAi was shown to further increase *ACTA2* expression in TGF-β-stimulated IPF fibroblasts, whereas *HDAC9* silencing decreased TGF-β-induced *ACTA2* expression in these cells [246]. Other studies showed that overexpression of HDAC9 and of its alternatively spliced isoform histone deacetylase-related protein (HDRP) in normal lung fibroblasts led to myofibroblast transformation and increased apoptosis resistance of transgenic fibroblasts [297], and HDRP was found in IHC studies to be significantly overexpressed in the myofibroblast foci of IPF lungs [165].

However, the role of the eminent upregulation of all four Class IIA HDACs in bronchiolar basal cells in IPF remains elusive and needs further investigation.

Interestingly, in contrast to Class I, HDAC inhibitors targeting specifically Class IIA HDACs (e.g., TMP269, MC1575 and MC1568) are poorly described in the literature, and there is yet no report about the use of Class IIA HDAC-inhibitors in the preclinical models of lung fibrosis. A study by Mannaerts et al. (2013) demonstrated that Class IIA HDAC inhibition by MC1568 blocked the activation of primary mouse hepatic stellate cells (HSC), as shown by the reduced expression of ECM genes *Col1a1*, *Col3a1*, *Acta2* and *Lox* [298]. Interestingly, this effect was mediated by the upregulation of miR-29 expression, which is an antifibrotic miRNA known to downregulate ECM molecules [87]. Moreover, knockdown of *Hdac4* by siRNA also resulted in significant miR-29 upregulation, *Col1a1* downregulation and partial inhibition of HSC activation [298]. In aggregate, this study revealed that HDAC4 regulates miR-29 expression, which represents a function of nuclear HDAC4 in fibrotic mesenchymal cells.

Due to a lack of studies about the use of Class IIA HDAC-inhibitors in preclinical models of lung fibrosis, published studies about the function, contribution and therapeutic correction of increased HDAC4 and -7 activity in fibrotic lung fibroblasts are yet based on single-gene silencing experiments and on the use of pan-HDAC inhibitors, as mentioned above. The effects of gene-targeting siRNAs for *HDAC4* and *HDAC7* are summarised in Table 3.

### 2.3. Class IIB Histone Deacetylases in IPF: Expression Profile, Function and Preclinical Studies

Immunohistochemical studies on IPF and normal control lung tissues revealed that both Class IIB HDAC enzymes HDAC6 and HDAC10 were found to be robustly upregulated in myofibroblasts within fibroblast foci in IPF. In contrast, expression of HDAC6 and -10 was absent in the interstitium of normal lungs. In accordance, immunoblot analyses of primary fibroblasts confirmed the upregulation of both Class IIB HDACs in IPF fibroblasts, and α-tubulin deacetylation, a surrogate marker for HDAC6 activity, was significantly increased in primary IPF versus normal lung fibroblasts [165]. Increased HDAC6 expression and consecutive α-tubulin-deacetylation have also been encountered in normal lung fibroblasts in response to TGF-β exposure [235], suggesting a crucial role of HDAC6 in TGF-β-dependent fibrogenesis. Other studies have shown that HDAC6 mediates TGF-β-induced EMT via SMAD3 activation in A549 cells, which was accompanied by α-tubulin-deacetylation and mesenchymal stress fibre formation [190,191,299]. Rapid HDAC6-dependent deacetylation of HSP90 was also observed, and a post-translational protein modification of HSP90 was reported to increase HSP90 chaperone function, which led to Notch1 activation during TGF-β-induced EMT [191]. Importantly, silencing of *HDAC6* by siRNA abrogated Notch1 signalling, decreased generation of EMT markers and restored expression of epithelial genes in TGF-β-treated A549 cells, indicating that HDAC6 was required for mediating the TGF-β–Notch1 signalling cascade during EMT [190,191]. Surprisingly, in fibrotic lung fibroblasts, siRNA-mediated silencing of *HDAC6* did not affect TGF-β-induced α-SMA and collagen-I expression. The same result was also observed for *HDAC10* silencing as well as for *HDAC6*/*HDAC10* double-knockdown [235]. These findings are interesting, as increased tubulin acetylation upon *HDAC6* silencing was observed, an indicator for successful HDAC6 inactivation. Thus, the cellular effects of its marked upregulation upon TGF-β in lung fibroblasts remain elusive and need further investigation. In addition, “HDAC6 gain of function” studies in human lung fibroblasts would help to clarify the role of overexpressed HDAC6 in IPF fibroblasts. This also applies to HDAC10, which has not yet been described much in lung fibrosis.

#### 2.3.1. Peculiar Role of HDAC6 in IPF Epithelial Cells?

**Figure 2:**
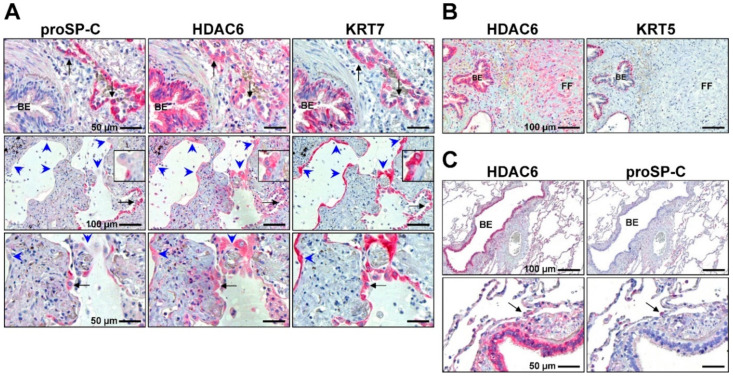
**Expression and localisation of HDAC6 in IPF lungs and normal control lungs.** (**A**) Representative immunohistochemistry (IHC) for proSP-C (AECII marker), HDAC6 and cytokeratin-7 (KRT7, marker for simple epithelia) in IPF lungs. Robust expression of HDAC6 was observed in AECII (proSP-C^+^ KRT7^+^, indicated by arrows) as well as hyperplastic AEC-like cells lining the alveoli (proSP-C^-^ KRT7^+^, indicated by blue arrowheads) and in bronchial epithelium (BE). (**B**) Robust expression of HDAC6 in fibroblast foci (FF) as well as KRT5^+^ bronchiolar basal cells and ciliated bronchial cells. (**C**) Representative IHC for proSP-C and HDAC6 in normal control lungs. HDAC6 was expressed in ciliated bronchial epithelium but not in AECII or normal lungs. Faint HDAC6 immunostaining was observed in the interstitium of normal lungs. Taken with permission from the study by Korfei et al. (2015) [165] (supplement), with modifications.

As a regulator of microtubule acetylation, HDAC6 is constitutively expressed in ciliated bronchial cells of normal lungs to regulate autophagy and mucociliary clearance [300]. In IPF, HDAC6 was found to be robustly upregulated in abnormal, hyperplastic bronchiolar structures, including airway basal cells (Figure 2) [165,174]. Subsequent loss-of-function studies revealed that HDAC6 appears to be responsible for the hyperproliferative phenotype of basal cells in IPF. It was thus suggested that overexpressed HDAC6 substantially governs the aberrant bronchiolization process in IPF [174].

Further, in contrast to all other HDAC enzymes, HDAC6 appeared to be the only HDAC expressed in IPF–AECII but not in the AECII of normal lungs (Figure 2) [165]. Because HDAC6 is involved in the aggresome formation and autophagic clearance of protein aggregates in response to increased misfolding (and impaired degradative capacity of the proteasome) [187], its induction in IPF–AECII was presumably caused by severe ER stress [18,20]. HDAC6 overexpression may represent an attempt of IPF–AECIIs to survive under conditions of irremediable ER stress but appear to be less able to counteract ER-stress-induced cell death. As described above, HDAC6 has also been widely reported as an eminent mediator of TGF-β-induced EMT in vitro, but AECIIs, as well as other epithelial cells, are not considered as cells giving rise to myofibroblasts in human IPF in vivo [40].

On the other hand, we could observe in our study that HDAC6 was also overexpressed in hyperplastic AEC cells without proSP-C expression (Figure 2) [165], which might suggest a role of HDAC6 in the differentiation of AECII to AECI or other AEC-like cells or to non-AEC epithelial cells. Interestingly, it has been reported very recently that human (but not murine) AECII transdifferentiate into metaplastic KRT5^+^-expressing basal cells in response to fibrotic signalling in the lung mesenchyme in *h*AECII-derived organoids ex vivo and in *h*AECII xenotransplantation experiments with bleomycin-treated mice in vivo [301]. Although speculative, the upregulation of HDAC6 in AEC, as well as bronchiolar basal cells in IPF lungs versus normal lungs, could suggest an involvement of this HDAC enzyme in the reprogramming of *h*AECII towards metaplastic basal cells, which have been widely observed to colonise alveolar spaces in close proximity to AECII in IPF [52,118,302]. The crucial involvement of HDAC6 in Notch1 pathway activation and transformation of epithelial cells supports this speculation.

#### 2.3.2. HDAC6 Selective Inhibitors in Preclinical Models of Lung Fibrosis

In contrast to all other Class II HDACs, isoform-selective inhibitors have been developed for HDAC6 and not only evaluated in cancer but also lung fibrosis (Table 4) [174,190,191,235].

Similar to silencing of *HDAC6*, pharmacological inhibition of HDAC6 deacetylase acitivity by the small molecule inhibitor tubacin resulted in the abrogation of TGF-β-induced Notch1 signalling and EMT in A549 cells [190,191]. However, studies with tubacin were not undertaken in TGF-β-stimulated lung fibroblasts but with the other selective HDAC6-inhibitor tubastatin. In contrast to genetic knockdown of *HDAC6*, tubastatin was shown to lead to α-tubulin hyperacetylation (and thus successful HDAC6 inhibition) and to repress TGF-β-induced expression of type-I collagen (*COL1A1*) in lung fibroblasts by inducing AKT dephosphorylation at Ser43 through increasing the association of AKT with the specific phosphatase PHLPP (PH domain and leucine-rich repeat protein phosphatase), with consequent AKT inactivation [235]. In addition, the expression of downstream targets of the PI3K-AKT pathway, such as HIF-1α and VEGF, was repressed by tubastatin. In contrast, TGF-β activated SMAD2/3 signalling as well as p38MAPK and ERK pathways were not affected by tubastatin. Further results of this study indicated the significant amelioration of bleomycin-induced lung fibrosis in mice in vivo by tubastatin treatment [235].

However, homozygous *Hdac6*^(−/−)^ knockout mice were not protected against bleomycin-induced lung fibrosis despite pronounced hyperacetylation of α-tubulin. Similarly, TGF-β-induced collagen expression was not decreased in murine lung fibroblasts isolated from *Hdac6*^(−/−)^ knockout mice compared to TGF-β-stimulated normal fibroblasts from wild-type mice. As mentioned above, RNAi-mediated knockout of *HDAC6* in human lung fibroblasts did also not repress TGF-β-induced collagen expression. Together, these results suggested that tubastatin might have ameliorated bleomycin-induced lung fibrosis by targeting the PI3K-AKT pathway, likely through an HDAC6-independent mechanism, and that tubastatin might have significant off-target effects aside from HDAC6 [235]. On the other hand, besides its protein deacetylase activity, HDAC6 has also been reported to exhibit a significant ubiquitin-binding capability and sequester ubiquitylated protein aggregates into autophagosomes or aggresomes [187]. Thus, the authors of this very interesting study also suggested that, simply, the loss or inhibition of HDAC6′s deacetylating function attenuated bleomycin-induced lung fibrosis by hyperacetylating target proteins, whereas in *Hdac6*^(−/−)^ knockout mice, the additional loss of HDAC6 function to transport cytotoxic polyubiquitinated (misfolded) proteins into autophagosomes for their degradation, may aggravate bleomycin-induced lung fibrosis by causing defective autophagy [235].

Very recently, novel specific inhibitors for human HDAC6 enzyme have been developed as promising pharmaceutical tools for the treatment of IPF, with the aim of also investigating the role of HDAC6 in the abnormal bronchiolization process in IPF, as this enzyme isoform was found to be robustly overexpressed in bronchiolar basal cells of IPF lungs [174]. It could be shown that the newly developed, specific HDAC6 inhibitor “compound 6h” reduced basal cell proliferation and bronchosphere formation in 3D organoid cultures derived from airway basal cells of IPF patients. In addition, compound 6h significantly inhibited TGF-β dependent fibrogenesis in cultured human lung tissues ex vivo, as shown by diminished expression of ECM genes *ACTA2*, *COL1A1*, *COL3A1* and *FN*. The authors of this study concluded that HDAC6 confers the pronounced hyperproliferative and profibrotic effects to bronchiolar basal cells in IPF, thereby underscoring that inhibition of HDAC6′s deacetylating function plays an important role in the treatment of IPF [174]. However, the effects of “compound 6h” on various non-canonical pathways activated by TGF-β were not evaluated in this study.

Taken together, HDAC6 deacetylase activity appears to mediate profibrotic responses and signalling through non-histone protein deacetylation, leading to altered cellular signalling, presumably involving HSP90 chaperone (a substrate of HDAC6)-mediated signalling and sustained AKT activation, which can be blocked by HDAC6 selective inhibitors. The selectivity of tubastatin to inhibit HDAC6 deacetylase activity should be re-checked.

## 3. Discussion of HDAC Inhibitors as Therapeutic Option for IPF

The summarised data provides evidence that abnormally increased HDAC activity in lung fibroblasts and bronchiolar basal cells versus a lack of HDAC activity in AECII is critical in the pathogenesis of lung fibrosis, which can be overcome by treatment with HDAC inhibitors. In particular, compelling evidence reveals a favourable therapeutic efficacy of pan-HDAC inhibitors in preclinical models of lung fibrosis (Table 1). Pan-HDAC inhibition through panobinostat inhibited FMD by reducing numerous ECM- and anti-apoptosis-related genes in IPF fibroblasts, while epigenetically repressed antifibrotic genes were restored by this drug. These beneficial effects were mediated largely through chromatin hyperacetylation and mechanisms involving non-histone protein acetylation and abrogation of p-STAT3 signalling [162,165,247,249]. In addition to the inactivation of Class I HDAC activity, panobinostat strongly inhibited HDAC6 activity (and also, therefore, with high probability, its profibrotic signalling) in IPF fibroblasts [249], which is in accordance with studies of its marvellous efficacy in various cancers [303]. Panobinostat also induced ER stress and proapoptotic signalling and thus led to the efficient inactivation of IPF fibroblasts [165]. Therefore, it is not surprising that a head-to-head comparison of the therapeutic effects of panobinostat versus the IPF drug pirfenidone has demonstrated the superior functionality of this pan-HDAC inhibitor over pirfenidone in acting against IPF-derived fibroblasts [249].

By employing an image-based screening assay with the use of α-SMA immunofluorescence intensity as the primary readout parameter of in vitro fibroblast activation (induced by TGF-β), Jones and coworkers (2019) tested and rank-ordered 99 modulators of epigenetic-regulating enzymes and identified the pan-HDAC inhibitor pracrinostat as the most effective small molecule in downregulating TGF-β-induced α-SMA expression [246]. Interestingly, panobinostat and SAHA were not included in this screening, but the first-discovered pan-HDAC inhibitor TSA (which is structurally related to SAHA) was evaluated and was 11th among the 99 compounds tested. Subsequent validation of pracrinostat revealed that it attenuated FMD through the derepression of the antifibrotic gene *PGC1A* and the suppression of various cytokine and ECM genes in TGF-β-stimulated IPF fibroblasts, which was, in part, attributed to the abrogation of HDAC7-mediated TGF-β signalling [246]. However, despite their beneficial effects on IPF fibroblasts, panobinostat and pracrinostat have yet not been evaluated in animal models of lung fibrosis in vivo. Additionally, two studies demonstrated the amelioration of pulmonary fibrosis and improved lung function in response to global HDAC inhibition by SAHA in bleomycin-treated C57Bl/6 mice in vivo [164,167]. The lung histopathology and health status of saline-treated control mice were not affected by SAHA [164]. SAHA was also shown to significantly attenuate paraquat-induced lung fibrosis in rats [252]. These findings indicate that hydroxamic-acid-based pan-HDAC inhibitors are well tolerated under conditions of lung fibrosis in vivo.

In IPF fibroblasts in vitro, SAHA suppressed genes associated with ECM and anti-apoptosis and upregulated proapoptotic genes through the modulation of chromatin acetylation and specific histone modifications associated with such genes and induced significant apoptosis in these cells [164,167]. In contrast, cell death was much less in normal control fibroblasts treated with SAHA [164]. This was similar to previously published studies showing that SAHA selectively induced malignant/tumour cells to undergo apoptosis but not normal cells [304]. This effect is important for the therapeutic efficacy of SAHA and tolerability in human patients. As mentioned in this article, panobinostat, pracrinostat and SAHA are FDA-approved drugs for cancer treatment but not the first-described pan-HDAC inhibitor TSA. Anyway, TSA was also shown in two studies to reduce the evolvement of lung fibrosis in bleomycin-treated rodents [168,251]. In fibrotic fibroblasts, it derepressed *FAS* expression [163] and abrogated TGF-β-induced AKT phosphorylation with consequent suppression of *ACTA2* and *COL1A1* expression [250].

As outlined in Section 2.1., some Class I-specific HDAC inhibitors, in particular the FDA-approved drugs VPA and romidepsin, also indicated a significant therapeutic effect in preclinical models of lung fibrosis in vitro and in vivo (Table 2), suggesting that targeting Class I HDACs is effective enough to abolish lung fibrosis. In support of this notion, HDAC1, -2 and -3 contribute to fibroblast anti-apoptosis through p53 inactivation as well as through epigenetic repression of proapoptotic genes via chromatin remodelling [163]. Further, Class I HDACs contribute to profibrotic signalling through the epigenetic silencing of antifibrotic genes [162,247] and through their involvement in various fibrotic signalling pathways, including SMAD2/3 (HDAC1) [252], PI3K (HDAC3) [265], ERK (HDAC3) [265], and JAK2/p-STAT3 (HDAC1, -2, -3) [249,262,263,264]. Moreover, Class I HDAC inhibitors, including the anti-epileptic drug VPA, have been shown to enhance fibrinolytic capacity. Although not shown in the setting of lung fibrosis, VPA was demonstrated to upregulate the expression of tissue plasminogen activator (t-PA/*PLAT*) in the vasculature of mice and men while downregulating PAI-1 [305,306]. The stimulatory effect of VPA on t-PA expression was associated with increased acetylation at the *PLAT* promoter [307]. VPA also reduced fibrin deposition and thrombus formation in mice after mechanical vessel injury but was not associated with an increased risk of bleeding [305]. In human patients with coronary disease, VPA increased the capacity for endogenous t-PA release and decreased plasma PAI-1 antigen [308]. Considering these studies and the reported antifibrotic effects of VPA on lung fibrosis in vitro and in vivo (Table 2) and the fact that fibrinolytic activity is impaired in lung fibrosis, VPA could also exert beneficial therapeutic effects in patients with IPF. This also applies to romidepsin, as it demonstrated significant inhibition of fibrosis development in the mouse model of bleomycin-induced lung fibrosis and as it revealed anti-fibrotic effects in vitro and in vivo at low nanomolar concentrations [169].

Recently, a novel clinical-stage HDAC inhibitor, CG-745, specific for Class I HDACs and Class IIB HDAC6, revealed favourable therapeutic efficacy in bleomycin-treated mice as it significantly reduced inflammatory cell populations in BALF and lowered the collagen contents back to the levels of the control saline group. Interestingly, CG-745 also efficiently attenuated severe lung fibrosis in mice induced by polyhexamethylene guanidine (PHMG) [233].

However, in vitro and experimental studies for lung fibrosis/IPF described in this review were mainly restricted to the assessment of inflammation status and fibroblast/myofibroblast apoptosis, but not on the effects of HDAC inhibitors on AECII injury/AECII death and the aberrant bronchiolar re-epithelialization process. Although nearly all Class I and Class II HDAC enzymes appeared to be actually absent in differentiated proSP-C^+^ IPF-AECII but abnormally upregulated in KRT5^+^ bronchiolar basal cells [165], it can be speculated that hyperplastic dedifferentiated AEC-like cells without proSP-C expression, as recently described as transitional KRT8^+high^ progenitors in fibrotic lungs [301,309,310], might overexpress HDAC enzymes for their terminal differentiation into KRT5^+^ basal cells. Interestingly, the administration of pan-HDAC inhibitors did not exert “deleterious” effects on fibrotic AECII in various rodent models of lung fibrosis but changed their abnormal phenotype. In the mouse model of bleomycin-induced lung fibrosis, Ota and coworkers (2015) showed that administration of the pan-HDAC inhibitor TSA from day 7 to 21 after bleomycin instillation restored *Sftpc* expression in FACS-isolated AECII in vivo [168]. Thus, although pan-HDAC inhibitors were shown to reduce lung fibrosis through the induction of significant myofibroblast apoptosis in bleomycin-treated mice, they appeared to reverse the aberrant hyperplastic (cytokine-releasing) phenotype of fibrotic AECII as well as to spare injured pro-apoptotic AECII from further apoptosis [164,168], thereby targeting two different AECII states in the fibrotic lung to promote proSP-C^+^ AECII re-differentiation, proSP-C^+^ AECII survival and proper re-epithelialization of the damaged alveolar epithelium as a therapeutic strategy. In renal fibrosis, it could be demonstrated in the murine model of unilateral ureteral obstruction that TSA led simultaneously to the inactivation of renal interstitial fibroblasts and the inhibition of renal tubular epithelial cell death [311]. However, the supposably beneficial effects of pan-HDAC inhibitors on AECII injury in experimental models of lung fibrosis as well as IPF *per se* are still underexplored.

Interestingly, a recent in vitro study showed that the weak pan-HDAC inhibitor 4-phenyl-butyrate (4-PBA) alleviated the aggregation and improved the secretion of IPF-associated mutant SP-A2 proteins in A549 cells through the upregulation of glucose-regulated protein (GRP)78 [255]. 4-PBA is an FDA-approved drug for the treatment of urea cycle disorders, and it is also well-known as a chemical chaperone exerting the proper folding of malfolded (mutant) proteins and the suppression of protein aggregation [224]. The study above suggests that the chaperone-like activity of 4-PBA may be (in part) mediated by its HDAC-inhibitor-function to upregulate genes involved in protein folding, but this remains to be elucidated. 4-PBA also attenuated bleomycin-induced lung fibrosis in rodents through the suppression of oxidative stress, NFκB activation and ER stress-mediated EMT induced by bleomycin [254,256] (Table 1).

However, the clinical use of pan-HDAC inhibitors in cancer patients has been, in part, associated with several challenges and side effects in some patients [312,313,314] that might be due to their broad activity across numerous HDAC isoforms and, thus, the concurrent inactivation of multiple HDAC family members, including their individual signalling. Significant side effects were also, in part, observed with romidepsin, suggesting that strong inhibition of more than one Class I isoform may be harmful to general cellular metabolism. On the other side, reports from clinical trials for NSCLC (non-small lung cancer) revealed that romidepsin and pan-HDAC inhibitors were well tolerated in patients [315]. Hence, the side effects are probably different depending on the genetic background as well as on the age of the patient. It might also be possible that lower doses of Class I/pan-HDAC inhibitors will be required in an IPF application compared to cancer.

The discussions about the side effects and improvement of therapeutic strategies have led to the development of HDAC isoform-selective inhibitors to overcome undesired effects [316]. Until now, most of the agents developed have selectivity for HDAC3, HDAC6 and HDAC8 and have been or are still currently evaluated in preclinical studies for cancer and lung fibrosis [171,189,234,235,236,237,238,258]. However, numerous HDACs have the very same targets. For example, HDACs 1, -2, -3 and -4 are well-known to be involved in the repression of *CIP1*^p21^ expression, and the knockdown of each of these HDACs resulted in the derepression of *CIP1*^p21^ expression. However, in each case, the magnitude of *CIP1*^p21^ induction was markedly less than that induced by pan-HDAC inhibitors [198]. In agreement, some studies clearly indicated that the magnitude of growth inhibition and apoptosis induced upon selective HDAC3 inhibition in tumour cells was relatively modest compared to the effects induced by Class I or pan-HDAC inhibitors [317,318]. On the other hand, selective inhibition of HDAC3, HDAC6 or HDAC8 has recently been demonstrated to exert remarkable therapeutic effects in the bleomycin mouse model of lung fibrosis [171,235,258]. However, the integrity of isoform-selective inhibitors mentioned above is not yet evidently proven, and these still could have off-target effects on many cellular pathways. Further, the novel HDAC6 selective inhibitor “compound 6h” [174] remains to be evaluated in experimental fibrosis.

**Figure 3:**
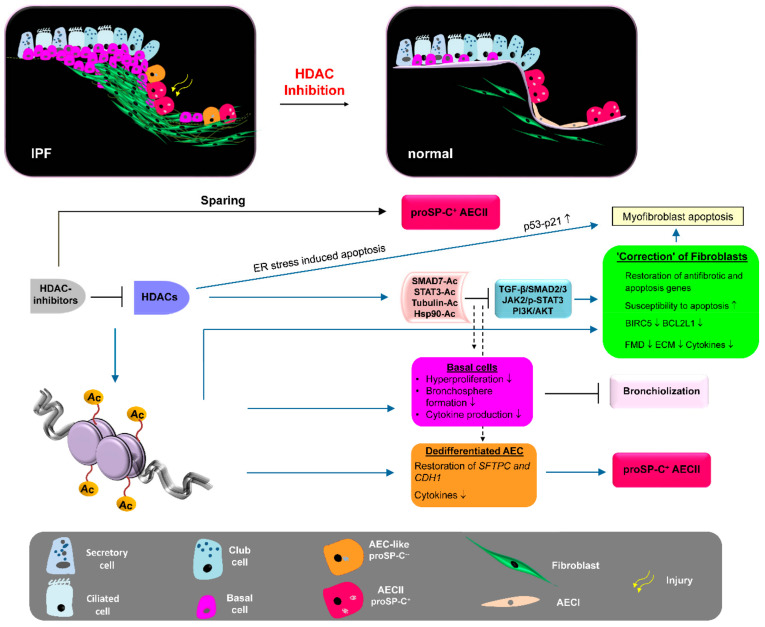
**Summary of putative therapeutic effects of HDAC-inhibitor treatment on IPF.** For details, see discussion. Abbreviations: IPF: idiopathic pulmonary fibrosis; ECM: extracellular matrix; FMD: fibroblast-to-myofibroblast differentiation; AECI/II: type-I/-II alveolar epithelial cell; Ac = acetylation; ↑: upregulation; ↓: downregulation.

Taken together, it can be suggested that specific inhibition of single HDACs may yield some therapeutic benefit, whereas the use of pan-HDAC inhibitors is likely to yield a stronger therapeutic response. In conclusion, the published findings summarised in this review indicate that HDACs offer novel molecular targets for IPF therapy and that FDA-approved Class I and pan-HDAC inhibitors for cancer treatment may also be promising therapeutic agents for the treatment of IPF. The putative antifibrotic effects of HDAC-inhibitor treatment on IPF are illustrated in Figure 3.

## 4. Conclusions and Future Perspectives

Idiopathic pulmonary fibrosis is associated with a progressive loss of lung function and a poor prognosis. Loss of AECII, myofibroblast expansion and the ectopic appearance of basal cells in the alveoli are the hallmarks of IPF. The number of myofibroblast foci, as well as the extent of alveolar KRT5^+^ basal cells, directly correlate with mortality in IPF [175,319]. Approved antifibrotic drugs, nintedanib and pirfenidone, modify disease progression, but IPF remains incurable, and there is an urgent need for new therapies. The majority of the evidence generated to date indicates that the overexpression of Class I and Class II HDACs is associated with fibroblast proliferation and FMD, as well as accounts for the apoptosis-resistant, invasive phenotype of fibroblasts/myofibroblasts and bronchiolar basal cells in IPF. Consistent with such a role, preclinical studies have shown that various Class I and pan-HDAC inhibitors not only reduced profibrotic signalling and ECM production but also stimulated growth arrest and cell death through the p53-p21 pathway and/or ER stress-induced apoptosis in fibrotic fibroblasts/myofibroblasts [164,165,169,271], a prerequisite for resolution of organ fibrosis, while AECII in fibrotic lungs were apparently spared from HDAC-inhibitor-induced apoptosis. Despite the proven antifibrotic efficacy of FDA-approved HDAC inhibitors in preclinical models, none of them have been approved for fibrotic diseases yet but could be readily progressed into an IPF clinical trial.

We believe that pan-HDAC inhibitors can not only reverse the aberrant epigenetic response in (myo)fibroblasts and bronchiolar basal cells in IPF but also the activated senescent phenotype in the AECII of IPF lungs despite pro-apoptotic events and a lack of many HDACs in IPF–AECII. The mechanisms of HDAC inhibitors towards the injured alveolar epithelium (including the aberrant epithelial repair mechanisms) in IPF have not yet been addressed and should be elucidated in future studies.

## Figures and Tables

**Table 1:** Pan-HDAC-inhibitors for treatment of pulmonary fibrosis/IPF.

Study	Lung Fibrosis Model	HDAC Inhibiton	Effect/Involved Molecules
Coward et al. (2009) [162]	TGF-β-treated IPF fibroblasts	*Panobinostat (LBH589)* pan-HDAC	H3 and H4 acetylation at *COX2* promoter, derepression of *COX2* expression
Huang et al. (2013) [163]	Lung fibroblasts of bleomycin mice, Primary IPF fibroblasts	*Trichostatin A (TSA)*, *vorinostat (SAHA)* pan-HDAC	H3 acetylation at the *Fas*/*FAS* promoter, derepression of *Fas*/*FAS* expression
Sanders et al. (2014) [164]	Primary IPF fibroblasts, Bleomycin mouse model	*Vorinostat (SAHA)* pan-HDAC	In vitro: proliferation H3 and H4 acetylation, H3K9Ac ↑ *BAK* ↑ *BID* ↑ *BCL2L1* ↓ In vivo: ameliorated lung fibrosis H3K9Ac ↑, *Bak* ↑ *Bcl2l1* ↓
Korfei et al. (2015) [165]	Primary IPF fibroblasts	*Panobinostat (LBH589)* pan-HDAC	Tubulin acetylation ↑, H3K27 acetylation, *CIP1*/p21 ↑, CHOP ↑, proliferation (*CCND1*) ↓, FMD (*ACTA2*, *COL1A1*, *COL1A3*, *FN*) ↓, surviving ↓ BCL-XL ↓
Zhang et al. (2013) [167]	Primary IPF fibroblasts, Bleomycin mouse model	*Vorinostat* *(SAHA)* pan-HDAC	In vitro: H3 and H4 acetylation, *COL3A1* (mRNA and protein) ↓ In vivo: ameliorated lung fibrosis, collagen-III ↓
Ota et al. (2015) [168]	TGF-β-stimulated A549 cells, Bleomycin mouse model	*Trichostatin A* *(TSA)*	In vitro: EMT ↓ restoration of *CDH1* expression. In vivo: Partial attenuation of fibrosis, restoration of AECII-*Sftpc* expression
Kim et al. (2019) [233]	Bleomycin mouse model, PHMG induced lung fibrosis	*CG-745* Class I-HDAC + HDAC6	Abrogation of bleomycin-fibrosis, H3 acetylation, Pai-1 ↓ α-Sma ↓ collagen-I ↓ BALF: Tnf-α ↓ Il-6 ↓ Attenuation of PHMG-fibrosis
Jones et al. (2019) [246]	TGF-β-treated primary IPF fibroblasts	*Pracrinostat* pan-HDAC, except HDAC6	H3 acetylation at *PGC1A* promoter, derepression of *PGC1A* expression, HDAC7 signalling ↓, *ACTA2* ↓ *TNC* ↓ *IL6* ↓ *PDGFA* ↓ Inhibition of FMD
Coward et al. (2010) [247]	TGF-β-treated primary IPF fibroblasts	*Panobinostat (LBH589)* pan-HDAC	H3 and H4 acetylation at *CXCL10* promoter, reduction of repressive H3K9Me3 at *CXCL10* promoter, derepression of *CXCL10* expression
Sanders et al. (2011) [248]	Fibrotic rat Thy1 (-) lung fibroblasts	*Trichostatin A* *(TSA)*	H3 and H4 acetylation, derepression of *Thy1* (*CD90*) expression
Korfei et al. (2018) [249]	Primary IPF fibroblasts	*Panobinostat (LBH589)* pan-HDAC	Tubulin acetylation ↑, H3K27Ac ↑ STAT3-pTyr705 ↓, proliferation ↓, FMD ↓ ECM (pro-collagen-I) ↓ HDAC1 ↓ HDAC2 ↓ HDAC7 (mRNA and protein) ↓
Guo et al. (2009) [250]	TGF-β-treated human normal lung fibroblasts	*Trichostatin A* *(TSA)* pan-HDAC	HDAC4 signalling ↓ *ACTA2* ↓ *COL1A1* ↓ *CTGF* ↑ (!) Inhibition of FMD, α-SMA ↓ AKT phosphorylation ↓
Ye et al. (2014) [251]	Bleomycin rat model	*Trichostatin A (TSA)* pan-HDAC	Reduction of lung fibrosis, *HDAC2* (mRNA and protein) ↓
Rao et al. (2016) [252]	TGF-β-treated normal human lung fibroblasts (HFL1), paraquat-induced lung fibrosis in rats	*Vorinostat* *(SAHA)* pan-HDAC	In vitro and in vivo: SMAD7 acetylation and stabilization, SMAD3 dephosphorylation, FMD ↓ attenuation of lung fibrosis
Glenisson et al. (2007) [253]	TGF-β-treated primary normal skin fibroblasts (human)	*Trichostatin A* *(TSA)* pan-HDAC	HDAC4 signalling ↓ *ACTA2*/α-SMA ↓ Inhibition of FMD
Kabel et al. (2016) [254]	Bleomycin rat model	*4-phenyl-butyrate* *(4-PBA)* Class I- and Class IIA-HDAC	attenuation of lung fibrosis, oxidative stress ↓ BALF: IL6 ↓ TGF-β ↓ TNF-α ↓
Jiang et al. (2018) [255]	A549 cells overexpressing mutant SP-A^G231V^ or SP-A^F198S^	*(4-PBA)* Class I- and Class IIA-HDAC	GRP78 ↑ suppressed protein aggregation, improved secretion
Zhao et al. (2015) [256]	Bleomycin mouse model	*(4-PBA)* Class I- and Class IIA-HDAC	ER stress ↓ EMT ↓ NK-κB (p65) ↓ cytokines ↓ α-SMA ↓ Col1a1 ↓ Col1a2 ↓ alleviation of lung fibrosis

Definition of abbreviations: IPF: idiopathic pulmonary fibrosis; EMT: epithelial–mesenchymal transition; ECM: extracellular matrix; FMD: fibroblast-to-myofibroblast differentiation; H3/H4: histone H3/H4; Ac: acetylation; BALF: bronchoalveolar lavage fluid; PHMG: polyhexamethylene guanidine; ↑: upregulation; ↓: downregulation.

**Table 2:** Class I HDAC inhibitors and Class I isoform-selective inhibitors for treatment of pulmonary fibrosis.

Study	Lung Fibrosis Model	HDAC Inhibition	Effect/Involved Molecules
Korfei et al. (2015) [165]	Primary IPF fibroblasts	*Valproic acid* *(VPA)* HDAC1, -2	H3K27 acetylation, CIP1 ↑, cell proliferation ↓ *BIRC5*/surviving ↓ collagen-I ↓ HDAC2 ↓ HDAC7 ↓
Conforti et al. (2017) [169]	TGF-β-treated primary IPF fibroblasts, Bleomycin mouse model	*Romidepsin* *(FK228)* HDAC1, -2	In vitro: H3 acetylation, *CIP1* ↑ *ACTA2* ↓ *COL3A1* ↓ *LOX* ↓ *HDAC4* ↓ inhibition of FMD. In vivo: inhibited lung fibrosis, ECM genes ↓ Lox protein ↓
Chen et al. (2021) [170]	TGF-β-treated A549 cells, Bleomycin mouse model	*Valproic acid* HDAC1, -2	In vitro and in vivo: SMAD2/3 deactivation, inhibition of EMT
Barter et al. (2010) [265]	TGF-β-treated embryonic mouse fibroblasts	*Entinostat* *(MS-275)* HDAC1, -3; *HDAC3* siRNA	Inhibition of PI3K and ERK pathways *Adam12* ↓ *Timp1* ↓
Davies et al. (2012) [275]	TGF-β-treated primary IPF fibroblasts	*Spiruchostatin A* HDAC1, -2	H3 acetylation, CIP1 ↑ cell proliferation ↓ inhibition of FMD
Noguchi et al. (2015) [278]	TGF-β-treated A549 cells	*Valproic acid* HDAC1, -2	H3K27 acetylation, partial inhibition of EMT
Kabel et al. (2016) [254]	Bleomycin rat model	*Valproic acid* HDAC1, -2	attenuation of lung fibrosis, oxidative stress ↓ BALF: IL6 ↓ TGF-β ↓ TNF-α ↓
Kamio et al. (2017) [279]	TGF-β-treated normal human lung fibroblasts	*Entinostat* *(MS-275)*: HDAC1, -3	Restoration of *ARHGEF3*/XPLN (negative regulator of *SPARC*) ↑ SPARC ↓
Saito et al. (2019) [171]	TGF-β-treated normal human lung fibroblasts, Bleomycin mouse model	*NCC170* HDAC8	In vitro: H3K27 acetylation, restoration of *PPARG* expression, ECM protein production ↓ In vivo: inhibited lung fibrosis
Chen et al. (2021) [258]	Bleomycin mouse model	*RGFP966* HDAC3	H3 acetylation at the *Nrf2* promoter, restoration of *Nrf2* expression, E-cadherin expression ↑ ECM proteins ↓
Yuan et al. (2020) [273]	RA-ILD mouse model	*HDAC3* siRNA	miR-19a-3p ↑ IL17RA ↓ IL17/IL17RA signalling ↓ Inflammation ↓ attenuation of lung fibrosis

Definition of abbreviations: IPF: idiopathic pulmonary fibrosis; EMT: epithelial–mesenchymal transition; ECM: extracellular matrix; FMD: fibroblast-to-myofibroblast differentiation; H3: histone H3; RA-ILD: rheumatoid arthritis (RA) associated interstitial lung disease (ILD); siRNA: small interfering RNA; BALF: bronchoalveolar lavage fluid; ↑: upregulation; ↓: downregulation.

**Table 3:** Effects of Class IIA gene targeting siRNAs in fibrotic fibroblasts.

Study	Model	HDAC Inhibiton	Effect/Involved Molecules
Jones et al. (2019) [246]	TGF-β-treated primary IPF fibroblasts	*HDAC7* siRNA	*ACTA2* ↓ *CTGF* ↓ *NOX4* ↓ *HDAC2* ↓ *HDAC6* ↓ *HDAC8* ↓ *HDAC10* ↓ *HDAC9* ↑ Derepression of *PGC1A* expression
Guo et al. (2009) [250]	TGF-β-treated human normal lung fibroblasts	*HDAC4* siRNA	ACTA2 ↓ Inhibition of FMD, AKT phosphorylation ↓
Glenisson et al. (2007) [253]	TGF-β-treated primary normal skin fibroblasts (human)	*HDAC4* siRNA	*ACTA2*/α-SMA ↓ FMD ↓ Upregulation of TGIF1 and TGIF2 (= Inhibitors of TGF-β signalling)
Kang et al. (2018) [294]	TGF-β-treated fibroblasts isolated from PD plaque	*HDAC7* siRNA	Inhibition of SMAD2/3 activation, FMD ↓ ECM protein production ↓
Hua et al. (2021) [296]	Endothelin-treated human normal lung fibroblasts (WI-38)	*HDAC7* siRNA	Deacetylation of AP-1, AP-1 activity ↓ α-SMA ↓ CTGF ↓

Definition of abbreviations: IPF: idiopathic pulmonary fibrosis; ECM: extracellular matrix; FMD: fibroblast-to-myofibroblast differentiation; PD: Peyronie’s disease; siRNA: small interfering RNA; ↑: upregulation; ↓: downregulation.

**Table 4:** Class IIB isoform-selective inhibitors for treatment of pulmonary fibrosis.

Study	Lung Fibrosis Model	HDAC Inhibition	Effect/Involved Molecules
Campiani et al. (2021) [174]	Organoid cultures derived from IPF basal cells, Ex vivo human lung model of fibrosis	*“Compound 6h”* HDAC6	Basal cell proliferation ↓ Bronchosphere formation ↓ Tubulin acetylation ↑ TGF-β dependent ECM synthesis ↓
Shan et al. (2008) [190]	TGF-β-stimulated A549 cells	*Tubacin* HDAC6 *HDAC6* siRNA	Tubulin-hyperacetylation, restoration of E-cadherin, SMAD3 phosphorylation ↓ PAI1 ↓ COL1A1 ↓ inhibition of EMT
Deskin et al. (2016) [191]	TGF-β-stimulated A549 cells	*Tubacin* HDAC6 *HDAC6* siRNA	Abrogation of TGF-β induced Notch1 signalling (HEY1, HES1 ↓) Acetylation of HSP90 (Ac-K294) p38 pathway ↓
Saito et al. (2017) [235]	TGF-β-stimulated human normal lung fibroblasts, Bleomycin mouse model	*Tubastatin A* HDAC6	In vitro and in vivo: Tubulin hyperacetylation, inhibition of PI3K-AKT pathway, FMD ↓ ECM ↓ amelioration of lung fibrosis

Definition of abbreviations: IPF: idiopathic pulmonary fibrosis; EMT: epithelial–mesenchymal transition; ECM: extracellular matrix; FMD: fibroblast-to-myofibroblast differentiation; siRNA: small interfering RNA; ↑: upregulation; ↓: downregulation.

## Data Availability

Not applicable.
